# Tracking CDK1 Dynamics With a Novel Biosensor Mouse and Clinical Cohorts to Predict CDK4/6 Inhibitor Sensitivity in ER+ Breast Cancer

**DOI:** 10.1002/advs.77920

**Published:** 2026-10-11

**Authors:** Sarah Alexandrou, Kristine J. Fernandez, Min Li Huang, Ewan K. A. Millar, Claire Vennin, Leila Eshraghi, Tan Dat Timothy Lam, Victoria Lee, Nyah C. Brooks, Janett Stoehr, Cristian Guaman Mendez, Douglas Strathdee, David Stevenson, David Herrmann, Sandra O'Toole, Paul Timpson, Max Nobis, C. Elizabeth Caldon

**Affiliations:** ^1^ St Vincent's Clinical School, Faculty of Medicine UNSW Sydney Sydney New South Wales Australia; ^2^ Garvan Institute of Medical Research Sydney NSW Australia; ^3^ SydPath Department of Anatomical Pathology St Vincent's Hospital Sydney New South Wales Australia; ^4^ Department of Anatomical Pathology NSW Health Pathology, St George Hospital Sydney New South Wales Australia; ^5^ St George & Sutherland Campus, School of Clinical Medicine UNSW Sydney Sydney New South Wales Australia; ^6^ Cancer Research UK Scotland Institute Glasgow Scotland UK; ^7^ School of Medical Science, Faculty of Medicine and Health University of Sydney Sydney New South Wales Australia; ^8^ Manchester Cell‐Matrix Centre, Division of Cell Matrix Biology and Regenerative Medicine, School of Biological Sciences, Faculty of Biology Medicine and Health The University of Manchester Manchester UK

**Keywords:** biosensor mouse, breast cancer, CDK1, CDK4/6 inhibition, cell cycle, endocrine therapy

## Abstract

High‐risk estrogen receptor positive (ER+) early breast cancer (EBC) is managed with endocrine therapies and cyclin‐dependent kinase (CDK) 4/6 inhibitors, although no standard molecular markers clinically identify these tumors. CDK1 lies immediately downstream of CDK4/6 but has not been investigated as a prognostic marker for ER+ disease. Here, we integrate real‐time imaging of a novel Cdk1 activity biosensor mouse with clinical cohorts to establish CDK1 as a candidate biomarker of progression and therapeutic sensitivity. Using the MMTV‐PyMT;MMTV‐Cre;Cdk1‐FRET biosensor mouse, we visualized Cdk1 upregulation in situ during the transition to invasive ductal carcinoma (IDC). Clinically, we identified that pCDK1^T161^ is specifically upregulated in IDC, and that pCDK1^Y15^ is prognostic of poor relapse‐free survival in stage 1 disease. We further characterized CDK1 activity as part of a dynamic adaptive response to CDK4/6 inhibition: the CDK1/cyclin B1 complex was suppressed, then rebounded within 3 days. In the NeoPalAna trial, non‐responders incompletely suppressed *CDK1*. Palbociclib‐resistant cells recovered basal CDK1 in vitro, non‐responsive tumors exhibited elevated Cdk1 activity in vivo, and *CDK1* was elevated in CDK4/6 inhibitor‐resistant patient tumors. Together, our findings position CDK1 as a candidate biomarker for poor prognosis in early disease, and an indicator of adaptive responses and resistance to CDK4/6 inhibition.

## Graphical abstract

Cyclin‐dependent kinase (CDK) 1 activity is a dynamic marker of treatment response in estrogen receptor positive breast cancer. Patient cohorts reveal CDK1 activity increases during progression to invasive disease, where high expression correlates with poor survival. Using a novel Cdk1 biosensor mouse alongside in vitro models, we demonstrate that CDK1 activity is an effective readout for CDK4/6 inhibitor sensitivity.

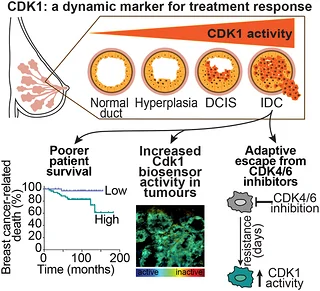

**Abbreviations**
AEC: Animal Ethics Committee
BrdU: bromodeoxyuridine
CDK: cyclin‐dependent kinase
CPTAC: Clinical Proteomic Tumor Analysis Consortium
DCIS: ductal carcinoma in situ
DEG: differentially expressed genes
DMSO: dimethyl sulfoxide
EBC: early breast cancer
ER‐: estrogen receptor negative
ER: estrogen receptor
ER+: estrogen receptor positive
FDR: false discovery rate
FLIM: fluorescence lifetime imaging microscopy
FRET: Förster resonance energy transfer
GEO: Gene Expression Omnibus
GFP: green fluorescent protein
GIMR: Garvan Institute of Medical Research
GMG: Garvan Molecular Genetics facility
HR: hazard ratio
IDC: invasive ductal carcinoma
IHC: immunohistochemistry
LSL: lox‐stop‐lox
MMTV‐PyMT: mouse mammary tumor virus‐polyoma middle T antigen
PalbR: palbociclib‐resistant MCF‐7 cells
PCR: polymerase chain reaction
PI: propidium iodide
px: pixel
RNA‐seq: RNA sequencing
ROC: receiver operating characteristic
ROI: region of interest
TCGA: The Cancer Genome Atlas

## 1 Introduction

Estrogen receptor positive (ER+) breast cancer is the most common subtype of breast cancer. While generally associated with a favorable prognosis, a significant subset of cancers present with high‐risk, aggressive features associated with recurrence and poor survival outcomes [1]. The management of high‐risk ER+ early breast cancer (EBC) has recently undergone a paradigm shift with the introduction of cyclin‐dependent kinase 4 and 6 (CDK4/6) inhibitors (ribociclib and abemaciclib) combined with endocrine therapy [2, 3]. In both *de novo* metastatic and metastatic settings, the combination of endocrine therapies (fulvestrant and aromatase inhibitors) and CDK4/6 inhibitors (palbociclib, ribociclib and abemaciclib) has been standard‐of‐care since 2015 [4, 5, 6]. This targeted approach arrests cancer cells at the G_1_/S checkpoint of the cell cycle, effectively blocking tumor proliferation.

Despite the clinical success of CDK4/6 inhibitors, identifying which patients will derive long‐term benefit remains a significant challenge. Current clinical practice relies on established markers such as estrogen receptor (ER) status, tumor size and grade, and lymph node involvement to stratify patients as high‐risk for recurrence. While a Ki67 index of >20% was initially a selection criterion for abemaciclib plus endocrine therapy for EBC, this requirement was subsequently removed [7]. Extended follow‐up demonstrated that Ki67 was not a definitive prognostic indicator for overall survival [3], and inconsistent inter‐laboratory reliability limits its clinical use [8].

While CDK4 and CDK6 are the targets of current therapeutic strategies, CDK1 is the central downstream regulator of cell division, controlling entry into mitosis. Unlike CDK2/4/6, CDK1 is the only non‐redundant and essential CDK that drives mitotic entry [9, 10]. Previous studies have implicated CDK1 overexpression in poor cancer outcomes [11, 12, 13, 14, 15, 16, 17], but its specific role as a dynamic indicator of the efficacy of CDK4/6 inhibitors has not been defined. While total mRNA and protein expression are useful indicators, the activity of CDK1 is precisely tuned by post‐translational phosphorylation. Specifically, the dynamic phosphorylation of CDK1 at the Y15 and T161 sites marks cell cycle progression. Monitoring the status of CDK1 phosphorylation provides a sensitive, functional readout of a cell's proliferative potential and its ability to bypass upstream cell cycle blockades.

In this study, we evaluated the prognostic and predictive significance of CDK1 in high‐risk EBC and recurrent ER+ breast cancer. We identified specific upregulation of CDK1 activity in early invasive disease. We further generated a Cdk1 activity biosensor mouse and crossed it to the MMTV‐PyMT mouse model to visualize Cdk1 activity in situ in real‐time, and analyzed samples from the NeoPalAna clinical trial to assess dynamic changes in *CDK1* during neoadjuvant palbociclib treatment. Our findings suggest that CDK1 activity has potential as a biomarker to identify high‐risk EBC, and that persistent CDK1 activity is a hallmark of treatment resistance and is a potential biomarker for identifying patients at risk of recurrence.

## 2 Materials and Methods

### 2.1 Cell Lines and Drug Treatment

MCF‐7 cells obtained from Michigan Cancer Foundation were cultured in phenol‐red‐free RPMI 1640 (Gibco) supplemented with 5% charcoal‐stripped fetal bovine serum, 1% penicillin/streptomycin (Invitrogen), and 10 pM 17β‐estradiol (E2758; Sigma‐Aldrich; resuspended in absolute ethanol). MCF‐7 cells were cultured for less than 6 months or 20 passages after short‐tandem repeat profiling (Garvan Molecular Genetics [GMG], Garvan Institute of Medical Research [GIMR]), and routinely tested negative for mycoplasma contamination. Cells were cultured under 5% CO_2_ in a humidified incubator at 37°C.

Cells were treated with 500 nM palbociclib (S1116; Selleckchem; resuspended in water), 1 µM ribociclib (HY‐15777; MedChemExpress; resuspended in dimethyl sulfoxide (DMSO)), 200 nM abemaciclib (HY‐16297; MedChemExpress; resuspended in water), or increasing concentrations of RO‐3306 (HY‐12529; MedChemExpress; resuspended in DMSO) for timeframes indicated in the figure legends.

Palbociclib‐resistant (PalbR) cells [18] were developed by continuously exposing MCF‐7 cells to 500 nM palbociclib for 10.3 months until a stable doubling time was achieved (Figure S5B). PalbR cells were subsequently maintained in 500 nM palbociclib, underwent short‐tandem repeat profiling (GMG, GIMR) where they were used for <20 passages post‐profiling, and tested negative for mycoplasma contamination.

### 2.2 Animal Work and Generation of the Cdk1‐FRET Biosensor Mouse

All animal experiments and endpoints were conducted in accordance with the St Vincent's Precinct and GIMR Animal Ethics Committee (AEC) guidelines (ARA 19/06, 19/10, 19/13, 22/08, 22/09, 22/10, 25/10, 25/11, 25/12) and the UK Animals (Scientific Procedures) Act 1986 (Project License P4B6898D0) and EU Directive 2010, and in compliance with the Australian Code of Practice for Care and Use of Animals for Scientific Purposes.

A conditional Cdk1‐FRET biosensor mouse was generated by targeting a lox‐stop‐lox (LSL) transgene under the control of the CAG promoter to the Hprt locus, as described previously [19, 20]. Briefly, the validated Cdk1‐FRET biosensor [21] was inserted into the pHprt.CAAG.STOP downstream of the LSL cassette and recombineered into the pSKB1 targeting vector [22]. The construct was introduced into Hprt‐deficient HM1 embryonic stem cells [23] by electroporation and cultured on a DR4 mouse embryonic fibroblast feeder layer. Following positive clone selection under HAT, a mouse line was derived by injection of mouse embryonic stem cells into C57BL/6J blastocysts in accordance with standard protocols. Germline transmission was confirmed by coat color and transgene verification by polymerase chain reaction (PCR) to mCerulean sequences.

Mouse mammary tumor virus‐polyoma middle T antigen (MMTV‐PyMT) mice [24] were crossed to an MMTV‐Cre mouse [25, 26] line and subsequently to the novel Cdk1‐FRET (Förster Resonance Energy Transfer) biosensor mouse model. All mice were bred at Australian BioResources or Biological Services Unit, and genotyping was performed at the GMG facility. Mice were kept in standard housing at a 12 h dark/light cycle and fed ad libitum.

Experimental mice were exclusively female and in the respective groups analyzed at 115 ± 30 days (wild‐type), 148 ± 53 days (hyperplasia), and 165 ± 29 days (invasive carcinoma) of age. Mice were randomly allocated to treatment groups once a cumulative tumor burden of 150–320 mm^3^ was reached and were 157 ± 43 days of age on average. For tamoxifen + ribociclib‐treated models, mice were implanted subcutaneously with slow‐release tamoxifen tablets (5 mg/60 day tablet; SE‐361; Innovative Research of America) and treated with 70 mg/kg ribociclib (S7440; Selleckchem) or vehicle (0.5% methylcellulose) by oral gavage five days a week once a cumulative tumor burden of 150–320 mm^3^ was reached. Animal numbers aligned with previous biosensor studies [27, 28]. Blinding was done in the final image analysis as described below.

### 2.3 Single‐cell Fluorescence Lifetime Imaging Microscopy (FLIM) Analysis

Excised tumors were imaged ex vivo for a maximum of 30–60 min post removal. Multi‐photon imaging was performed using a Leica DMI 6000 SP8 confocal microscope using a 25 × 0.95 NA water immersion objective on an inverted stage equipped with a Picoquant Multiharp. The Ti:Sapphire femtosecond laser (Coherent Chameleon Ultra II, Coherent) excitation source operating at 80 MHz was tuned to a pumping wavelength of 840 nm. An RLD‐HyD detector was used with a 483/40 nm bandpass emission filter in order to detect mCerulean. Images were acquired at a line rate of 700 Hz, 512 × 512 pixel (px) and at a total of 203 frames per image. Realignment of the data was performed using Galene (v2.0.2) [29] using the warp realignment mode, 10 realignment points, a smoothing radius of 2 px and a realignment threshold of 0.6 for the mCerulean signal. Single‐cell analysis was performed in a blinded manner using FLIMfit (v5.1.1) [30] by drawing a region of interest (ROI) of individual cells based on intensity images from the mCerulean channel. Subsequently, the global fitting routine is run by FLIMfit, and single‐cell lifetime in ns recorded.

### 2.4 Immunoprecipitation and Immunoblotting

Cell lysates were separated on 4%–12% Bis‐Tris gels (Invitrogen) as previously described [31, 32]. Immunoprecipitation was performed against 500 µg of cell lysate using protein A‐Sepharose beads (Invitrogen) and cyclin B1 (1:100 dilution; #12231; Cell Signaling Technology) antibody.

The following primary antibodies for immunoblotting were used at 1:1000 dilution: cyclin B1 (#4135; Cell Signaling Technology), cyclin B1 (ab181593; Abcam), CDK1 (ab18; Abcam) and pCDK1^T161^ (ab201008; Abcam). RB was used at a 1:500 dilution (#554136; BD Pharmingen) and GAPDH was used at 1:15000 dilution (sc‐32233; Santa Cruz Biotechnology). Membranes were probed with horseradish peroxidase‐conjugated donkey anti‐rabbit (NA934V; Cytiva) or sheep anti‐mouse (NA931V; Cytiva) secondary antibodies at a 1:1000 dilution, and proteins were visualized using Western Lighting Chemiluminescence reagent (PerkinElmer) on the Fusion FX7 imager (Bio‐Rad) or X‐ray film. Densitometric analysis was performed using FIJI/ImageJ software [33]. Plot profiles were generated using the ‘gels’ analysis tool. Peaks corresponding to protein bands were manually demarcated using the ‘straight line’ tool, and the ‘wand’ tool provided the densitometric measurement of the area under the peak.

### 2.5 Quantitative Real‐Time PCR Analysis

Total RNA was extracted from cell pellets and reverse transcription was performed as previously described [32]. *CDK1* (hs00938777_m1), *CCNB1* (hs01030099_m1) and *GAPDH* (Hs02758991_g1) Taqman probes (Thermo Fisher Scientific) were used to analyze mRNA expression levels using a QuantStudio7 Flex Real‐Time PCR System. Relative gene expression was assessed using the ΔΔ threshold cycles method [34], where expression was normalized to *GAPDH*.

### 2.6 DNA Replication Assay

S‐phase percentages were measured by flow cytometric analysis of bromodeoxyuridine (BrdU) incorporation in propidium iodide (PI)‐stained, ethanol‐fixed cells. Cells were pulsed with 10 µM BrdU (Sigma‐Aldrich) for 2 h. BrdU incorporation was detected with anti‐BrdU‐FITC (BD Biosciences) for 1 h, and cells were co‐stained with 1 µg/mL PI (Sigma‐Aldrich) and incubated with 0.5 mg/mL RNaseA as previously described [32]. Flow cytometry was performed on a FACSCantoII (BD Biosciences). At least 30,000 events per replicate were analyzed using FlowJo version 10.8.1 (BD Life Sciences).

### 2.7 Proliferation and Clonogenic Assay

Metabolic assays were performed using AlamarBlue (Thermo Fisher Scientific) on cells treated with RO‐3306 or DMSO (vehicle) for 3 days. IC_50_ values were determined by log‐transforming, normalizing, and plotting data with a nonlinear fit of least square analysis, as previously described [32].

Clonogenic assays were performed on PalbR cells maintained in 500 nM palbociclib and treated for 2 weeks with RO‐3306. Drugs were replenished every 3–4 days. Colonies were fixed with ice‐cold methanol and stained with 0.1% crystal violet (Sigma‐Aldrich). Plates were scanned and analyzed as previously described [32].

### 2.8 Clinical Cohorts

#### 2.8.1 Garvan/Royal Prince Alfred Hospital Breast Progression Series

This cohort comprises early pre‐invasive and invasive lesions from 222 patients diagnosed between 1996 and 2005 at Royal Prince Alfred Hospital, Sydney, Australia [35]. Ethics approval was granted by the Ethics Committee of the Royal Prince Alfred Hospital (X05‐0115). The Breast Progression Series includes tissue from 23 patients with at least 2 matched samples across hyperplasia, ductal carcinoma in situ (DCIS) and/or IDC.

#### 2.8.2 Invasive Ductal Cohort

This cohort consists of biopsies from 292 patients diagnosed with invasive ductal carcinoma (IDC) treated between February 1992 and August 2002 at St Vincent's Hospital, Sydney, Australia [36]. A detailed description of the clinicopathological characteristics of the cohort is published in [36, 37, 38]. Ethics approval was granted by the Human Research Ethics Committee of St Vincent's Hospital (HREC SVH H94/080 and HREC 06336 SVH H00036).

Tumors with low pCDK1^T161^ expression were defined as H‐score <20 and high expression defined as H‐score ≥20. This is based on (1) the observation that 20 is the median pCDK1^T161^ tumor H‐score in the Breast Progression Series for positive cases in pre‐invasive (hyperplasia and DCIS) lesions, and (2) analysis by receiver operating characteristic (ROC) curve and Youden's index [39]. Tumors with low CDK1 expression were defined as H‐score <66 and high expression defined as H‐score ≥66 based on ROC curve analysis and Youden's index [39]. The cut‐off for Ki67 was defined at 5% (the median expression level as previously published [38]).

#### 2.8.3 CDK1, pCDK1^T161^, Ki67 and Green Fluorescent Protein (GFP) Immunohistochemistry (IHC) and Quantification

Two tissue microarray cohorts were analyzed using IHC for CDK1 and pCDK1^T161^: the Garvan/Royal Prince Alfred Hospital Breast Progression Series (n = 222) [35], and an Invasive Ductal cohort (n = 292) [36]. Staining was performed using the BOND RX Automated stainer (Leica Biosystems). For CDK1 staining, heat‐induced epitope retrieval was performed at pH 6 (Bond Epitope Retrieval solution 1, Leica Biosystems), 100°C for 30 min, before antibody incubation (1:2000; ab18; Abcam) for 1 h. For pCDK1^T161^ staining, heat‐induced epitope retrieval was performed at pH 9 (Bond Epitope Retrieval solution 2, Leica Biosystems) before antibody incubation (1:2000; ab201008; Abcam) for 1 h. Detection was performed with diaminobenzidine, and slides were counterstained with hematoxylin. Slides were imaged using the AperioCS2 slide scanner (Leica Biosystems). CDK1 and pCDK1^T161^ staining was assessed blinded by pathologist M.L.H. (Breast Progression Series) or E.K.A.M. (Invasive Ductal cohort) using the H‐score system that sums the percentage of tumor cells for each staining intensity (0–3), giving a range of 0 to 300 [40].

Ki67 staining was previously performed on the Invasive Ductal cohort (n = 292) [36] as published in [38]. Staining was assessed as a percentage of Ki67‐positive cells.

Expression of the Cdk1 biosensor in mammary tissue was confirmed by staining for GFP as described previously [28] using the BOND RX Automated stainer. Heat‐induced epitope retrieval was performed at pH 9 (Bond Epitope Retrieval solution 2), 93°C for 30 min, before antibody incubation (1:500; A‐11122; Thermo Fisher Scientific) for 1 h. Slides were counterstained and cover slipped as described above. The stained slides were digitally scanned using the Hamamatsu NanoZoomer S210 Digital slide scanner and ROIs exported using QuPath (v0.5.1) [41].

### 2.9 RNA Sequencing (RNA‐seq)

Total RNA was extracted from MCF‐7 cells treated with 48 h of vehicle or 500 nM palbociclib, and PalbR cells maintained in 500 nM palbociclib as described above. RNA quality was assessed using the High Sensitivity RNA Screen Tape Assay (Agilent Technologies), and all samples processed for RNA‐seq had an RNA integrity number ≥ 9.8. RNA‐seq libraries were prepared using the KAPA mRNA HyperPrep kit (Roche) according to manufacturer's instructions. Reads were sequenced on a NovaSeq 6000 instrument (Illumina), using 150‐base‐pair paired‐end sequencing chemistry, by the Sequencing Platform (GIMR).

FASTQ files were quality‐checked by *FastQC* (v0.11.9) [42]. Sequence adaptors and quality filtering was performed using *trimmomatic* (v0.39.1) and processed paired‐end reads were aligned to the reference genome assembly ‘GRCh38_2020‐A_build’ [43] using *STAR* (v2.7.8) [44] with default parameters. Gene expression was quantified by counting the number of reads aligned to each Ensembl gene model using *featureCounts* (v2.0.1) [45], available through the package *RSubread* (https://bioconductor.org/packages/release/bioc/html/Rsubread.html).

Downstream statistical analysis was carried out in *R* (v4.3.1), and differentially expressed genes (DEGs) between the different treatment groups were identified using the *limma‐voom* method (v3.56.2) [46, 47]. Library size normalization was performed using the Trimmed Mean of M‐values method from *edgeR* (v3.42.4) [48]. Genes with low expression were filtered out prior to DEG analysis, and only genes with at least 1 count per million reads in the three biological replicates were kept (Table S1). Differential expression analysis was performed using empirical Bayes‐moderated t‐statistics within the *limma‐voom* framework. p‐values were adjusted for multiple testing using the Benjamini‐Hochberg method to control the false discovery rate (FDR), with FDR <0.05 considered statistically significant (Table S2). Genes that were upregulated or downregulated more than 1‐fold (log_2_FC) were analyzed for over‐representation of Hallmark gene sets using ShinyGo [49], which applies a hypergeometric test with FDR correction (Table S2).

### 2.10 In Silico Datasets

#### 2.10.1 The Cancer Genome Atlas (TCGA) and Clinical Proteomic Tumor Analysis Consortium (CPTAC) Breast Cancer Datasets

CDK1 phosphoprotein site expression (n = 66 primary breast cancers) was analyzed from CPTAC [50] data and obtained from cBioPortal [51, 52] in November 2025. Data was analyzed by two‐sided Pearson's correlation test.

Protein expression data for CDK1, pCDK1^T161^, pCDK1^Y15^, cyclin A2, cyclin B1 and Ki67 from normal breast and primary breast tissue were analyzed from the CPTAC [50] dataset. Data and statistical comparisons were accessed via UALCAN [53].

#### 2.10.2 Survival Analyses

Datasets of relapse‐free survival of breast cancer patients were accessed via KMplotter [54] with probes for CDK1_pY15, *CDK1* (*CDC2*; 203213_at), *CCNA2* (213226_at), *CCNB1* (228729_at), and *MKI67* (212023_s_at) analyzed. For CDK1_pY15, tumors were demarcated by tertiles into high (upper tertile) and low (lower 2 tertiles) expression. For composite *CDK1*&*cyclin* signatures, the mean expression of probes was used, and tumors split by median expression. For *MKI67*, tumors were split by median expression. Survival analyses were performed using Log‐rank (Mantel‐Cox) tests on GraphPad Prism. Hazard ratios (HR) (Log‐rank) were computed for each analysis.

#### 2.10.3 Neoadjuvant Palbociclib Treatment (NeoPalAna Cohort)

Array‐based gene expression and associated clinical data from NeoPalAna clinical trial [55] cohort of anastrozole and palbociclib‐treated ER+ breast cancers were downloaded from GSE93204 at the Gene Expression Omnibus (GEO) NCBI database. Tumors were stratified into responders and non‐responders based on Ki67 expression, with responders defined in the clinical trial as having <10% Ki67 after 4 cycles of palbociclib. Comparisons were performed by one‐way ANOVA with mixed effect analysis with Holm‐Šidák's multiple comparisons test.

#### 2.10.4 Metastatic ER+ Palbociclib + Endocrine Therapy‐Treated Cohort

Transcriptome data (normalized transcripts per million) of tumors from 23 patients with matched baseline biopsy and progressive disease samples was downloaded from GSE186901 at the GEO NCBI database [56]. Patients had metastatic disease (*de novo* or recurrent) at time of biopsy and received palbociclib with endocrine therapy (letrozole, fulvestrant or exemestane + GnRH agonist). The average time to progression was 10.9 months (1.5–25.2 months). Comparison of transcript levels of pre‐treatment and progressive disease was performed for *CDK1*, *CCNA2*, *CCNB1*, *CDK1/CCNA2* ratio, *CDK1/CCNB1* ratio, *MKI67* and *RB1*. Analysis performed by Wilcoxon matched‐paired signed‐rank test.

### 2.11 Statistical Analysis

Data were analyzed in GraphPad Prism (v.10), using two‐tailed t‐test, one‐way ANOVA, two‐sided non‐parametric Mann‐Whitney test or Wilcoxon matched‐paired signed‐rank test as appropriate. When one‐way ANOVA was used, the differences between individual groups were compared using Tukey's multiple comparisons test or Holm‐Šidák's multiple comparisons test, and Dunn's multiple comparison test was applied where Kruskal‐Wallis test was used. Pearson's analysis was performed to examine correlation between datasets. Kaplan‐Meier survival analysis was performed with the Log‐rank (Mantel‐Cox) test, and HR were calculated. The Cox proportional hazards model was used for multivariate analysis of breast cancer‐related death. Violin plots and box and whiskers plots are presented from minimum to maximum, with the range extending from the first to third quartile, and the line in the middle indicating the median. All in vitro experiments were performed in biological triplicate from independent passages, except where indicated. Error bars in each figure indicate mean ± standard error of the mean (SEM).

## 3 Results

### 3.1 CDK1 Activity Increases With Progression of Breast Tumorigenesis

To evaluate CDK1 as a candidate biomarker for high‐risk breast cancer, we investigated the earliest stages of breast cancer invasion. We modelled Cdk1 activity during early tumorigenesis by crossing the MMTV‐Cre driver line and the MMTV‐PyMT transgenic mouse model [57] with the Cdk1‐FRET biosensor mouse (Figure 1A and Figure S1A,B). This model recapitulates spontaneous mammary cancer, developing invasive carcinomas after 165 ± 29 days. Early lesions in this model are ER+, although ER expression can be lost in carcinomas and metastatic lesions [57]. Mice generated using the biosensor were assessed for expression of the biosensor using anti‐green fluorescent protein (GFP) IHC in the mammary gland and tumors (Figure S1C).

**FIGURE 1:**
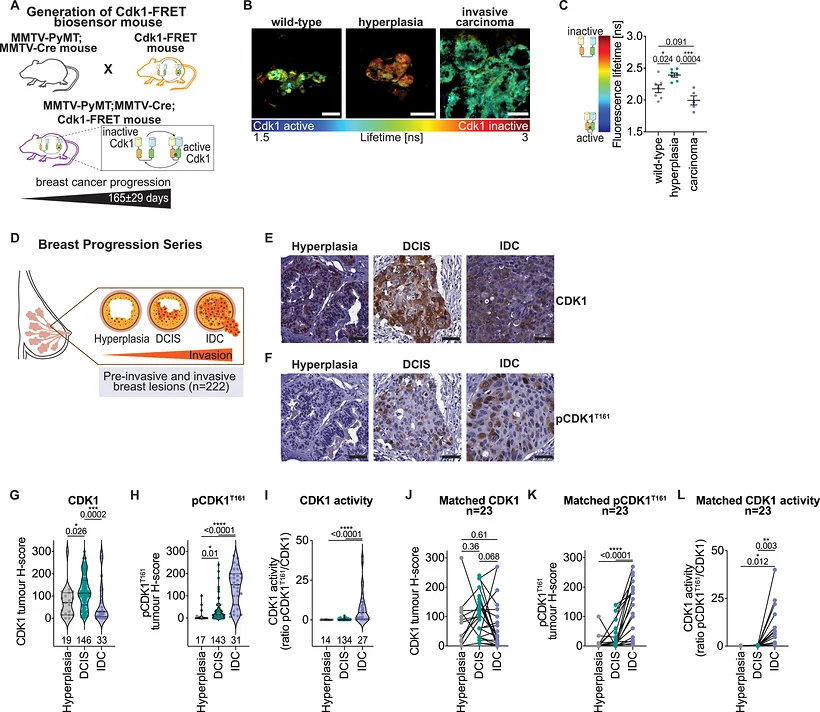
CDK1 activity increases as breast cancers become invasive. (A) Schematic of Cdk1‐FRET (Förster Resonance Energy Transfer) biosensor mouse crossed with the MMTV‐Cre driver line and the MMTV‐PyMT mouse model of breast cancer allowing for the quantification of Cdk1 activity at a single‐cell level. (B) Representative images of upregulated Cdk1 activity in primary MMTV‐PyMT;MMTV‐Cre‐driven mammary tumors compared to wild‐type and hyperplastic mammary lesions. (C) Quantification of Cdk1 activity from data in (B). Data analyzed by one‐way ANOVA with Tukey's multiple comparisons test (n = 5–7 mice per condition, 558 cells analyzed in total). (D) Schematic of the Breast Progression Series of 222 pre‐invasive (hyperplasia and ductal carcinoma in situ (DCIS)) and invasive (invasive ductal carcinoma (IDC)) lesions [35]. Representative immunohistochemistry (IHC) of (E) total CDK1 and (F) pCDK1^T161^ during hyperplasia, DCIS and IDC. IHC analysis of (G) total CDK1, (H) pCDK1^T161,^ and (I) CDK1 activity (calculated as the ratio of pCDK1^T161^ to total CDK1 expression) in hyperplasia, DCIS and IDC from available cores. Each point in (G‐I) represents one tumor; data were analyzed by Kruskal‐Wallis test with Dunn's multiple comparisons test. (J‐L) Analysis from 23 tumors with at least 2 matched samples of hyperplasia, DCIS or IDC stained for (J) total CDK1, (K) pCDK1^T161^ and (L) CDK1 activity (calculated as the ratio of pCDK1^T161^ to total CDK1 expression). Data in (J–L) analyzed by one‐way ANOVA with mixed effect analysis with Holm‐Šidák's multiple comparisons test. Scale bars in (B, E‐F) = 50 µm.

Using FLIM, we measured the lifetime of the donor fluorophore mCerulean within the intramolecular, reversible and highly validated Cdk1‐FRET reporter [21]. The Cdk1 reporter contains the 16‐amino‐acid autophosphorylation site of cyclin B1 which is exclusively phosphorylated in cells when the endogenous Cdk1/cyclin B1 complex is activated. The conformational change resulting from this Cdk1/cyclin B1 activation leads to a FRET conformation of the reporter correlating with Cdk1 activity within the cells as previously reported by us and others [21, 58]. This allowed for the assessment of Cdk1/cyclin B1 activity at the single‐cell level within the mammary gland and mammary tumors. In the colormaps, inactive Cdk1 (higher FLIM lifetime) is represented by red/yellow, while active Cdk1 (lower FLIM lifetime) is shown by green/blue (Figure 1B). Lifetime assessments were performed on single‐cell ROIs selected based on mammary architecture. Comparison of wild‐type mammary ducts with hyperplasia and invasive carcinoma revealed that Cdk1 activity is significantly reduced in mammary hyperplasia (average lifetime 2.39 ns) compared to wild‐type ducts (average lifetime 2.18 ns), but upon progression from hyperplasia to carcinoma, is significantly upregulated in MMTV‐PyMT‐driven carcinomas (average lifetime 2.0 ns; Figure 1C). In parallel, we performed IHC of Cdk1 and pCdk1^T161^, a Cdk1 activation site that is readily detected by IHC, on MMTV‐PyMT‐driven mammary lesions (Figure S1D). We observed a progressive increase in both Cdk1 and pCdk1^T161^ expression from wild‐type to hyperplasia and carcinoma. This suggests that Cdk1 activity is primed to increase upon progression to invasive disease.

Having established that the activity of Cdk1 is elevated in invasive carcinomas compared to pre‐invasive hyperplastic lesions, we next examined the association of total and phosphorylated CDK1 in a cohort of 222 untreated pre‐invasive and invasive breast lesions [35] (Figure 1D). We performed IHC on pre‐invasive and invasive breast lesions, for which 198 (CDK1) and 191 (pCDK1^T161^) cores were successfully stained. For both total CDK1 and pCDK1^T161^, we observed a mixed distribution of nuclear and cytoplasmic staining, consistent with established CDK1 dynamics where inactive protein accumulates in the cytoplasm prior to nuclear enrichment upon T161 activation (Figure 1E‐F) [59, 60]. IHC analysis showed that total CDK1 expression increased during the transition from pre‐invasive hyperplasia to DCIS; however, we observed a significant decrease in total CDK1 expression from DCIS to IDC (Figure 1G). Conversely, pCDK1^T161^ expression significantly increased from pre‐invasive hyperplasia and DCIS lesions to invasive carcinoma (Figure 1H), resulting in a net increase in active CDK1 (ratio pCDK1^T161^/CDK1) in IDC (Figure 1I).

Finally, we analyzed expression of CDK1 and pCDK1^T161^ in matched tissue of pre‐invasive and invasive lesions from 23 patients (Figure 1J–L). While there was no difference in total CDK1 expression across the different stages (Figure 1J), we observed significant increases in pCDK1^T161^ expression and CDK1 activity during the transition from hyperplasia or DCIS to IDC in patient‐matched samples (Figure 1K,L). Therefore, CDK1 activity increases during the progression to invasive disease, in both human cohorts and a mouse model of tumorigenesis.

### 3.2 CDK1 and Invasive Breast Cancer

Given that overexpression and activation of CDK1 have previously been observed in many cancer types [11, 12, 13, 14, 15, 16, 17], we investigated how its expression and activity are regulated in invasive breast cancer. We first profiled the relationship between total CDK1 and its phosphorylation sites (Y15 and T161) and breast cancer stage by examining the CPTAC breast cancer dataset [50]. While total CDK1 protein (Figure 2A) and active CDK1 (phospho‐T161) (Figure 2B) levels in EBC (stage 1) were comparable to normal breast tissue, possibly due to the small sample size, levels of pCDK1^Y15^ were significantly elevated in stage 1 disease (Figure 2C). Advanced disease (stages 2 and 3) was associated with widespread upregulation of total CDK1 and phosphorylated CDK1 (pCDK1^T161^ and pCDK1^Y15^) compared to normal tissue (Figure 2A–C) suggesting that while specific phosphorylation events mark early disease and progress through to late stages, total CDK1 upregulation is a feature of late‐stage disease.

**FIGURE 2:**
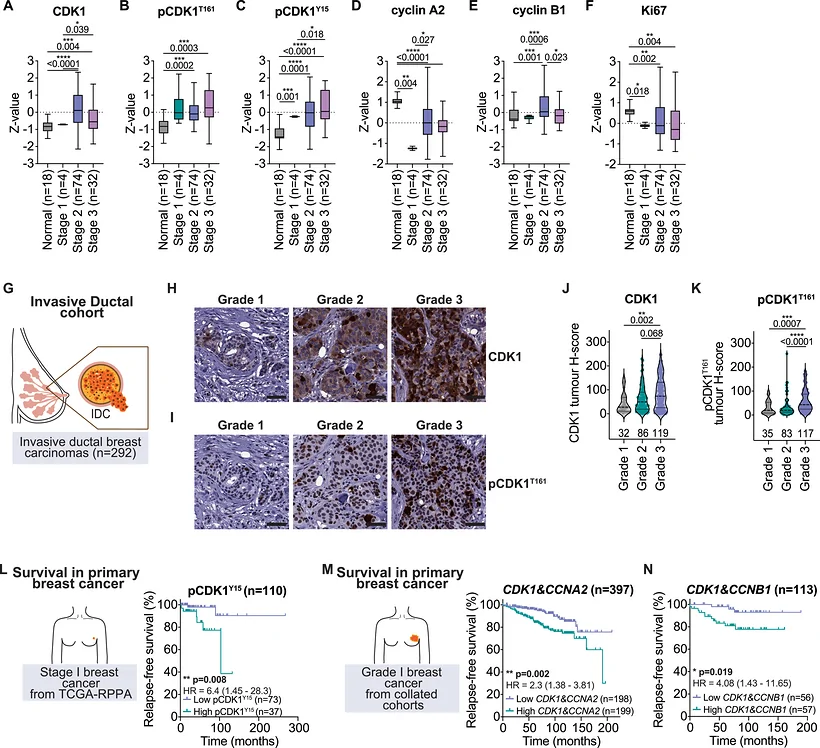
Invasive breast cancer has high CDK1 activity. Protein expression from the Clinical Proteomic Tumor Analysis Consortium [50] across normal and primary breast tissue separated by disease stage for (A) total CDK1, (B) pCDK1^T161^, (C) pCDK1^Y15^, (D) cyclin A2, (E) cyclin B1, and (F) Ki67. Data were analyzed as described previously in [53]. (G) Schematic of the Invasive Ductal cohort of 292 invasive ductal carcinomas (IDC) [36]. Representative immunohistochemistry (IHC) of (H) total CDK1 and (I) pCDK1^T161^ in grade 1, 2, and 3 IDC. Scale bar = 50 µm. IHC analysis of (J) total CDK1 and (K) pCDK1^T161^ in grade 1, 2 and 3 IDC from available cores. Each point in (J‐K) represents one patient; data analyzed by Kruskal‐Wallis test with Dunn's multiple comparisons test. Relapse‐free survival of patients with high and low (L) pCDK1^Y15^, (M) *CDK1&CCNA2*, and (N) *CDK1&CCNB1* in breast cancer patients with stage 1 (L) and grade 1 (M‐N) disease from multiple combined cohorts accessed via KMPlotter [54]. Data in (L–N) was analyzed by Log‐rank (Mantel‐Cox) test with hazard ratio.

We also examined the protein expression of CDK1's cyclin binding partners. Expression of cyclin A2 was significantly reduced across all breast cancer stages (stages 1–3) relative to normal tissue (Figure 2D). Cyclin B1 protein was significantly increased in stage 2 breast tumors but remained unchanged in stages 1 and 3 (Figure 2E). Notably, the expression profile of CDK1 was distinct from that of the common proliferation marker Ki67, which was significantly lower in stage 1, 2, and 3 breast tumor tissue compared to normal tissue (Figure 2F), indicating that CDK1 captures a cell cycle dynamic distinct from standard proliferation markers.

We next evaluated the expression of CDK1 and pCDK1^T161^ by IHC in a cohort of 292 IDCs (Figure 2G–I) [36] and benchmarked it against archival IHC for the established proliferation marker Ki67 [38]. Due to exhaustion of cores, we analyzed 237, 235, and 254 tumors for CDK1, pCDK1^T161,^ and Ki67 expression respectively. To explore how CDK1 tracks progression of invasive disease relative to Ki67, we compared expression levels of total CDK1, pCDK1^T161,^ and Ki67 across grade 1, 2, and 3 tumors (Figure 2H–K and Figure S2A). Total CDK1 expression increased significantly from grade 1 to grade 3 tumors (Figure 2J), while pCDK1^T161^ and Ki67 both increased significantly from grade 1 and grade 2 to grade 3 tumors (Figure 2K and Figure S2A). Together, these data show that CDK1 activity increases with tumor grade and that pCDK1^T161^ performs comparably to Ki67 as a readout of more advanced and aggressive stages of breast cancer.

We then assessed the prognostic importance of CDK1 in early‐stage breast cancer by examining collated publicly available breast cancer cohorts [54] (Figure 2L–N). Relapse‐free survival was significantly lower in patients with stage 1 breast cancer whose tumors had high pCDK1^Y15^ (p = 0.008; Figure 2L); survival data from Ki67 and pCDK1^T161^ were not available from the TCGA‐RPPA cohort for direct comparison. CDK1 is phosphorylated at specific stages of mitotic entry [60], and in breast cancers there is a significant positive correlation between the CDK1 phospho‐sites Y15 and T161 (*r* = 0.63; *p* < 0.0001) (Figure S2B). Furthermore, using a composite gene expression score of *CDK1* and its cyclin partners, we found that a high *CDK1&cyclin* signature was associated with worse relapse‐free survival in grade 1 disease compared to low expression (*CDK1&CCNA2*: *p* = 0.002; Figure 2M; *CDK1&CCNB1: p* = 0.019; Figure 2N). High *MKI67* was similarly associated with poor survival in grade 1 disease (*p* = 0.004; Figure S2C). In summary, these data identify CDK1 as a potent prognostic marker of relapse in early‐stage breast cancer, performing on par with Ki67.

### 3.3 CDK1 Expression Predicts Response to Endocrine Therapies and CDK4/6 Inhibitors, but Not Chemotherapy

Therapeutic strategies for breast cancer are dictated by subtype, with ER+ patients historically receiving endocrine‐directed therapies and estrogen receptor negative (ER‐) patients receiving chemotherapy regimens. To evaluate the predictive value of CDK1 alongside Ki67 in these therapeutic contexts, we stratified the cohort of 292 IDCs [36] (Figure 2G) by historic ER status. While total CDK1 protein expression was comparable between ER+ and ER‐ tumors (Figure S3A), ER‐ breast cancers had significantly higher levels of pCDK1^T161^ and Ki67 (Figure S3B,C), consistent with the more proliferative phenotype of ER‐ disease. Total and phosphorylated CDK1 showed a low but significant correlation in ER+ breast cancers (*r* = 0.46; *p* < 0.0001; Figure S3D) and a moderate but significant correlation in ER‐ tumors (*r* = 0.6; *p* < 0.0001; Figure S3E), indicating that CDK1 activation is differentially regulated between breast cancer subtypes. Ki67 and CDK1 showed comparable, significant correlations in ER+ and ER‐ breast cancer (*r* = 0.41 and *r* = 0.46, respectively; *p* < 0.0001; Figure S3F,G), supporting CDK1 as a proliferation readout that tracks Ki67 across ER subtypes.

We next examined the association of CDK1 and pCDK1^T161^, as detected by IHC (Figure 3A), with survival across endocrine therapy‐treated ER+ patients and chemotherapy‐treated ER‐ patients. High expression of total CDK1 and pCDK1^T161^ was predictive of breast cancer‐related death in patients with ER+ breast cancer treated with endocrine therapy (Figure 3B), whereas Ki67 was not (Figure S3H). By contrast, none of the markers were predictive of outcome for patients with ER‐ disease treated with standard‐of‐care chemotherapy (Figure S3I). In a multivariate analysis, neither CDK1 nor Ki67 were independent predictors for breast cancer‐related death after adjustment for lymph node status and ER status (Table 1). This parallels the well‐documented behavior of Ki67, whose independent prognostic value is similarly absent in multivariate analyses of comparable cohorts [61, 62]. While this positions CDK1 as a candidate proliferation biomarker of equivalent clinical utility to Ki67 in this setting, we further investigate its potential utility below in the more clinically relevant contexts of high‐risk early disease considered for CDK4/6 inhibitor treatment and endocrine therapy‐resistant disease that subsequently receives CDK4/6 inhibition.

**FIGURE 3:**
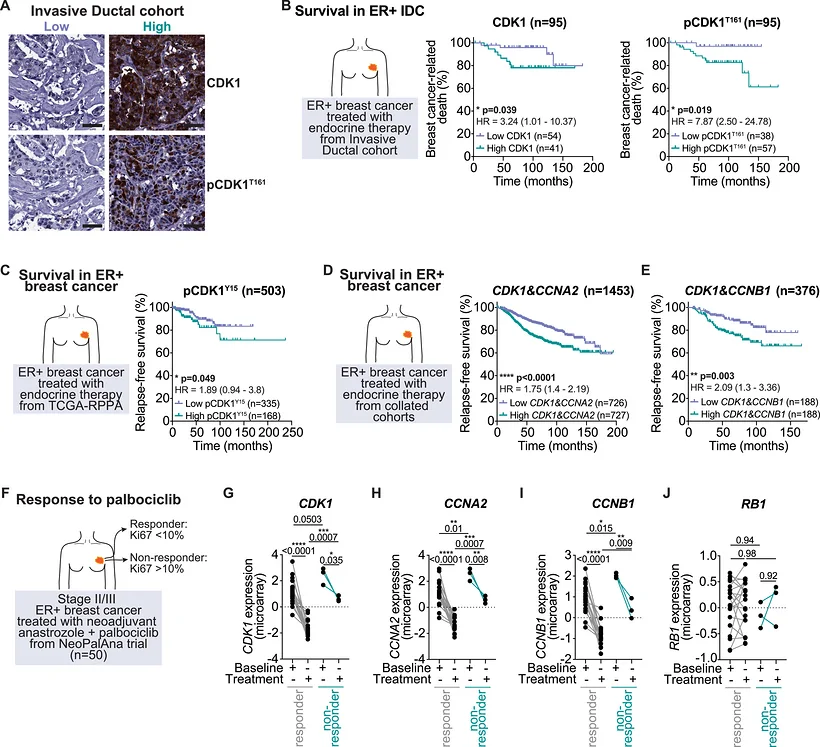
High CDK1 expression is associated with poor survival in treated estrogen receptor positive (ER+) breast cancer patients. (A) Representative immunohistochemistry (IHC) of high and low total CDK1 and pCDK1^T161^ from the Invasive Ductal cohort [36]. Scale bar = 50 µm. (B) Breast cancer‐related death in endocrine therapy‐treated ER+ invasive ductal carcinomas (IDC) split by high and low total CDK1 and pCDK1^T161^ from the Invasive Ductal cohort [36]. Relapse‐free survival of endocrine therapy‐treated ER+ breast cancers split by high and low (C) pCDK1^Y15^, (D) *CDK1&CCNA2*, and (E) *CDK1&CCNB1* from multiple combined cohorts accessed via KMPlotter [54]. Data in (B–E) analyzed by Log‐rank (Mantel‐Cox) test with hazard ratio. (F) Schematic of the NeoPalAna clinical trial of 50 ER+ breast cancer patients [55]. mRNA expression of (G) *CDK1*, (H) *CCNA2*, (I) *CCNB1* and (J) *RB1* in tumors of ER+ breast cancer patients with available baseline and palbociclib‐treated samples. Responders (*CDK1, CCNA2* and *RB1*: *n* = 19; *CCNB1*: n = 18) and non‐responders (*n* = 3) were stratified based on Ki67 expression. Comparisons between responders and non‐responders analyzed by one‐way ANOVA with mixed effect analysis with Holm‐Šidák's multiple comparisons test.

**TABLE 1:** Multivariate cox regression analysis of CDK1 and clinicopathological variables for breast cancer‐related death.

Variable	HR	95% CI	*p* value
Grade 3	1.78	0.82–3.94	0.145
Size >20 mm	1.15	0.64–2.13	0.648
Lymph node positive	3.25	1.68–6.66	0.0003
ER positive	0.47	0.23–0.96	0.04
Patient age	1.01	0.99–1.03	0.393
CDK1 expression	1.00	0.99–1.00	0.313
Ki67%	1.01	1.00–1.03	0.132

We validated our findings in a second dataset of ER+ and ER‐ breast cancers treated with standard‐of‐care therapy from collated publicly available breast cancer cohorts [54]. Relapse‐free survival was significantly lower in endocrine therapy‐treated ER+ tumors with high pCDK1^Y15^ (*p* = 0.049; Figure 3C). Conversely, there was no difference in survival based on pCDK1^Y15^ detection in ER‐ breast cancer samples from patients treated with chemotherapy (Figure S3J). Using composite gene expression scores, we found that high *CDK1&cyclin* signatures were associated with worse relapse‐free survival in ER+ breast cancer patients treated with endocrine therapy compared to low *CDK1&cyclin* expression (*CDK1&CCNA2*: *p* < 0.0001; Figure 3D; *CDK1&CCNB1: p* = 0.003; Figure 3E). High *MKI67* expression was also associated with poorer survival in this setting (*p* < 0.0001; Figure S3K). There was no difference in relapse‐free survival of chemotherapy‐treated ER‐ patients with *CDK1&cyclin* or *MKI67* expression (Figure S3L–N), reinforcing the potential utility of *CDK1&cyclin* signatures as predictive markers specifically for endocrine therapy‐treated ER+ breast cancer.

Finally, as the current standard‐of‐care for early‐stage high‐risk breast cancer involves the addition of CDK4/6 inhibitors to endocrine therapy [2, 3], we examined the association of *CDK1*, *CCNA2, CCNB1*, and *RB1* with treatment response. In the NeoPalAna clinical trial, patients (*n* = 50) underwent serial biopsies with intervening neoadjuvant anastrozole and palbociclib (Figure 3F) [55]. Response to treatment in the clinical trial was designated as Ki67<10% after 4 cycles of palbociclib. We found a trend of higher *CDK1* expression in non‐responders compared to responders (Figure 3G), and *CCNA2* and *CCNB1* expression was significantly higher in non‐responders (Figure 3H–I). Importantly, expression of these markers remained significantly elevated in non‐responders following treatment. *RB1* expression, by contrast, did not differ between responders and non‐responders (Figure 3J), despite *RB1* loss being a canonical CDK4/6 inhibitor resistance mechanism [63, 64]. These findings suggest that high residual activity of the CDK1/cyclin axis is an indicator of non‐response to CDK4/6 inhibition.

### 3.4 CDK1 Activity is a Marker of the Adaptive Response to CDK4/6 Inhibitors

Given that high *CDK1* and cyclin expression is detected in tumors of patients who are non‐responders to CDK4/6 inhibitors in the NeoPalAna trial, we next investigated the dynamic response of CDK1 to CDK4/6 inhibitors. We treated MCF‐7 cells with 500 nM palbociclib and assessed cell cycle progression over a 120 h time period. Quantification of total DNA content and BrdU incorporation (Figure 4A and Figure S4A) confirmed that palbociclib treatment resulted in a profound loss of cellular proliferation at 24–48 h (Figure 4B), but cell cycle progression began to increase at 72 h, although this was not significant. This is consistent with the previously observed adaptive response to CDK4/6 inhibitors where there is an initial cell cycle arrest followed by partial cell cycle re‐entry [65].

**FIGURE 4:**
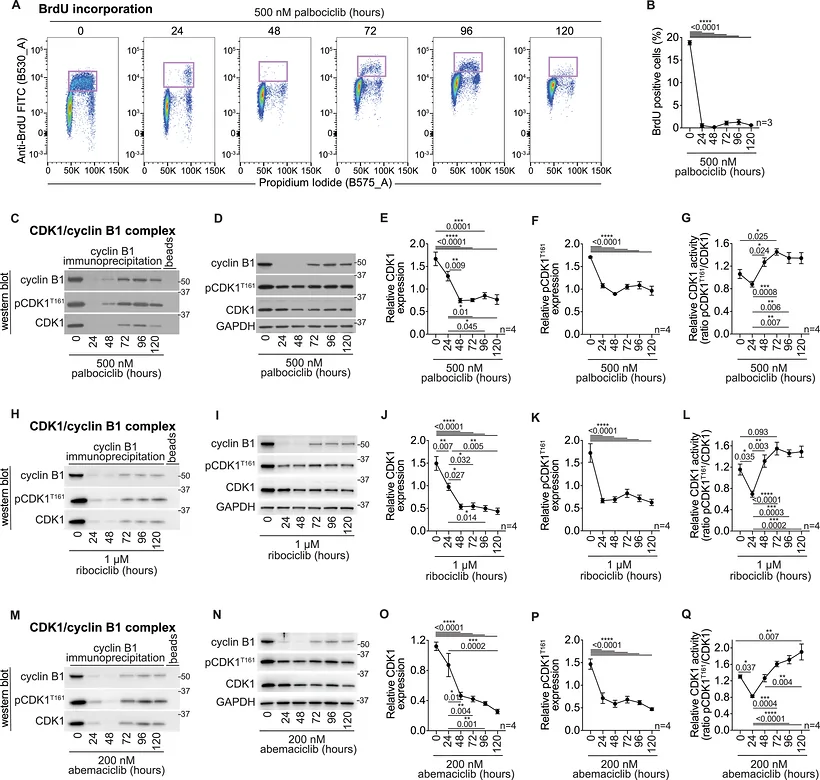
Short‐term response to CDK4/6 inhibition followed by adaptive upregulation of CDK1 activity. (A) Flow cytometric analysis of bromodeoxyuridine (BrdU) incorporation in MCF‐7 cells treated with 500 nM palbociclib over a 120 h time course. Representative flow cytometry plots from 3 biological replicates (with gating strategy in Supporting Information S1: Figure 4A). (B) Quantitation of BrdU‐positive cells from 3 biological replicates in (A). (C) Lysates from MCF‐7 cells treated with 500 nM palbociclib were immunoprecipitated with cyclin B1 and Western blotted for CDK1, pCDK1^T161^ and cyclin B1 (full‐length blots in Supporting Information S1: Figure 4B,C, where one membrane has been probed with all antibodies). Representative of 3 biological replicates. (D) Representative Western blots of CDK1, pCDK1^T161^, cyclin B1 and GAPDH in MCF‐7 cells treated with 500 nM palbociclib from 4 biological replicates (full‐length blots in Supporting Information S1: Figure 4F, where one membrane has been probed with all antibodies). Quantitation of (E) CDK1, (F) pCDK1^T161^ and (G) CDK1 activity (calculated as the ratio of pCDK1^T161^ to total CDK1 expression) by densitometry from 4 biological replicates. Band intensity normalized to respective GAPDH in (E‐F) or respective CDK1 expression in (G). (H) Lysates from MCF‐7 cells treated with 1 µM ribociclib were immunoprecipitated with cyclin B1 and Western blotted for CDK1, pCDK1^T161,^ and cyclin B1 (full‐length blots in Supporting Information S1: Figure 4H‐I, where one membrane has been probed with all antibodies). Representative of 3 biological replicates. (I) Representative Western blots of CDK1, pCDK1^T161^, cyclin B1 and GAPDH in MCF‐7 cells treated with 1 µM ribociclib from 4 biological replicates (full‐length blots in Supporting Information S1: Figure 4J, where one membrane has been probed with all antibodies). Quantitation of (J) CDK1, (K) pCDK1^T161^ and (L) CDK1 activity (calculated as the ratio of pCDK1^T161^ to total CDK1 expression) by densitometry from 4 biological replicates. Band intensity normalized to respective GAPDH in (J‐K) or respective CDK1 expression in (L). (M) Lysates from MCF‐7 cells treated with 200 nM abemaciclib were immunoprecipitated with cyclin B1 and Western blotted for CDK1, pCDK1^T161^ and cyclin B1 (full length blots in Supporting Information S1: Figure 4L,M, where one membrane has been probed with all antibodies). Representative of 3 biological replicates. (N) Representative Western blots of CDK1, pCDK1^T161^, cyclin B1 and GAPDH in MCF‐7 cells treated with 200 nM abemaciclib from 4 biological replicates (full length blots in Supporting Information S1: Figure 4N, where one membrane has been probed with all antibodies). Quantitation of (O) CDK1, (P) pCDK1^T161,^ and (Q) CDK1 activity (calculated as the ratio of pCDK1^T161^ to total CDK1 expression) by densitometry from 4 biological replicates. Band intensity normalized to respective GAPDH in (O‐P) or respective CDK1 expression in (Q). Data in (B, E–G, J–L, O–Q) analyzed by one‐way ANOVA with Tukey's multiple comparisons test, error bars = standard error of the mean.

We then examined the abundance of active CDK1/cyclin B1 complexes during the treatment time course (Figure 4C and Figure S4B,C). Following treatment with palbociclib, the CDK1/cyclin B1 complex could not be detected at 24 h. However, from 48 h the CDK1/cyclin B1 complex could be detected and increased through to 120 h, coinciding with low‐level proliferation (Figure 4C). We also examined the levels of unbound total and phosphorylated CDK1 in palbociclib‐treated MCF‐7 cells and noted that unbound CDK1 was in excess of cyclin B1 in both asynchronous and palbociclib‐inhibited cells (Figure S4C).

We also investigated how the mitotic CDK1/cyclin B1 axis was affected during the adaptive response to CDK4/6 inhibitor treatment. *CDK1* and *CCNB1* mRNA expression were significantly decreased within 24–48 h. However, from 72 h, expression of *CDK1* and *CCNB1* were upregulated, although they did not return to basal levels by 120 h (Figure S4D,E). We observed similar changes in protein during the adaptive response (Figure 4D and Figure S4F). Palbociclib treatment led to a significant reduction in CDK1, pCDK1^T161,^ and cyclin B1 expression after 48 h (Figure 4E,F and Figure S4G). However, from 72 h there was a rebound of CDK1 activity (the ratio of pCDK1^T161^/CDK1) to higher than basal levels (Figure 4G). Taken together, these data indicate that despite a G_1_ arrest and reduced total protein level, the relative specific activity of the CDK1/cyclin B1 complex is adaptively upregulated during short‐term CDK4/6 inhibition.

To determine whether this adaptive upregulation of CDK1 activity is a class effect rather than palbociclib‐specific, we repeated the immunoprecipitation and Western blotting experiments using two additional clinically approved CDK4/6 inhibitors: ribociclib and abemaciclib. Both drugs produced the same pattern as palbociclib: the CDK1/cyclin B1 complex was undetectable at 24 h and re‐established from 48 h (ribociclib: Figure 4H and Figure S4H,I and abemaciclib: Figure 4M and Figure S4L,M). CDK1, pCDK1^T161^ and cyclin B1 protein levels were significantly reduced by 48 h (ribociclib: Figures 4I‐K and Figure S4J,K and abemaciclib: Figures 4N‐P and Figure S4N,O); and CDK1 activity rebounded to (ribociclib) and above (abemaciclib) basal levels from 72 h (ribociclib: Figure 4L and abemaciclib: Figure 4Q). Together, these data indicate that adaptive upregulation of CDK1/cyclin B1 activity is a shared response across the CDK4/6 inhibitor class.

### 3.5 Palbociclib‐resistant Cells Retain CDK1 Expression

While CDK4/6 inhibitors are effective in the short‐term (Figure 4A), the development of drug resistance is inevitable [66]. To investigate the role of CDK1 in this long‐term adaptation, we established an in vitro model of acquired CDK4/6 inhibitor resistance by culturing MCF‐7 cells in 500 nM palbociclib for approximately 10 months (Figure 5A) [18]. As the cells acquired resistance to palbociclib (Figure S5A), the doubling time decreased to a constant rate of ∼56 h (Figure 5B and Figure S5B). While this represents a recovery of proliferation, the resistant cells remained significantly slower than MCF‐7‐Vehicle cells (∼34 h; *p* < 0.0001; Figure S5B), despite maintaining RB expression (Figure S5C,D).

**FIGURE 5:**
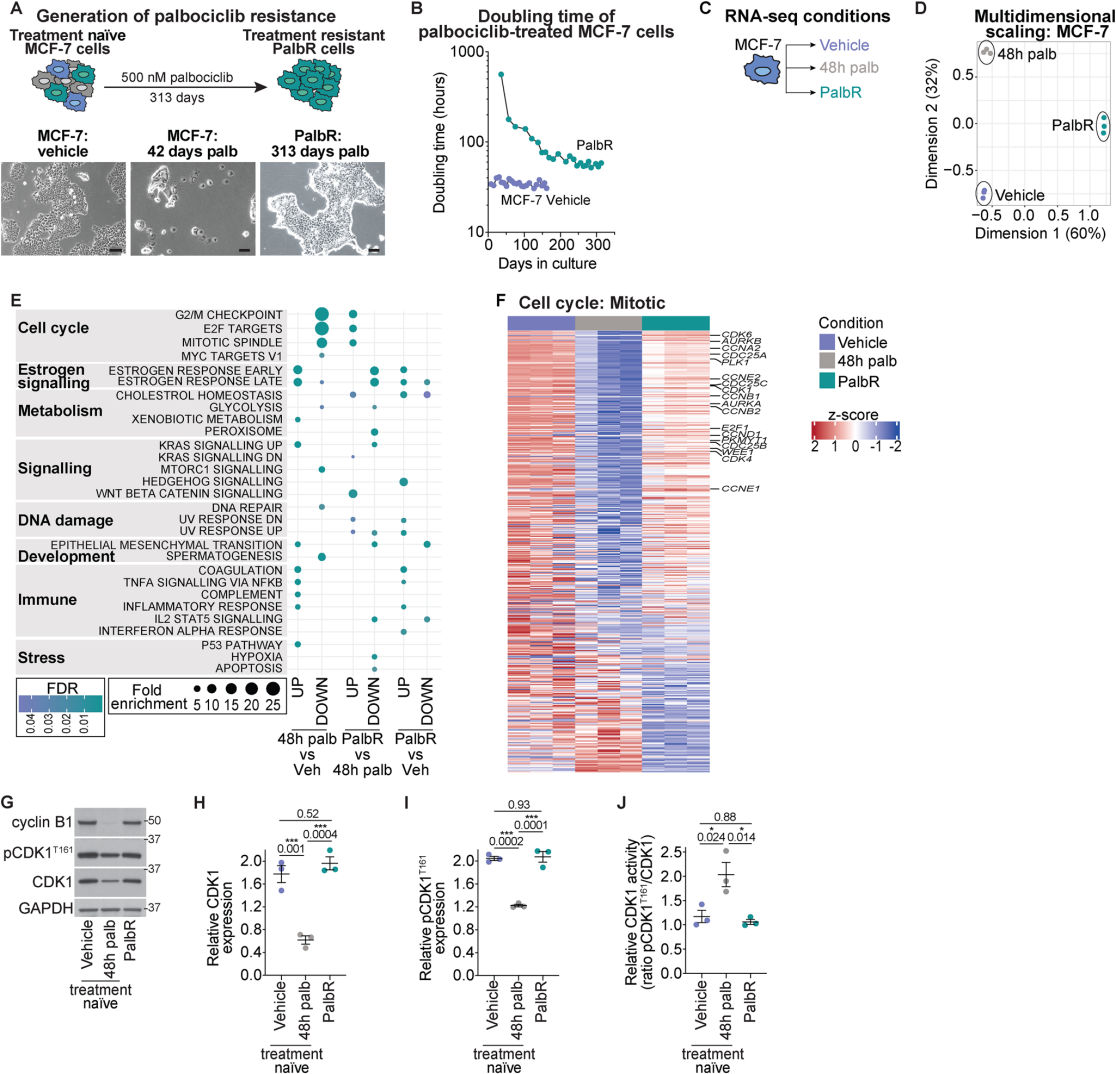
Palbociclib‐resistant MCF‐7 cells restore CDK1 expression and activity. (A) Schematic of the in vitro generation of palbociclib‐resistant estrogen receptor positive (ER+) MCF‐7 breast cancer cells (PalbR). Phase contrast images show phenotypic changes during acquisition of palbociclib resistance. Scale bar = 100 µm. (B) Doubling time of PalbR and passage‐matched Vehicle cell lines. (C) RNA‐seq experimental schematic. MCF‐7 cells were treated with vehicle or 500 nM palbociclib (palb) for 48 h, alongside PalbR cells. (D) Multidimensional scaling analysis of RNA‐seq data of MCF‐7 Vehicle, 48 h palbociclib (palb) and PalbR cells from 3 biological replicates. (E) Pathway enrichment analysis of Hallmark gene sets. (F) Heatmap of genes in the REACTOME_CELL_CYCLE_MITOTIC gene set. (G) Representative Western blots of CDK1, pCDK1^T161^, cyclin B1 and GAPDH in Vehicle‐treated and 48 h 500 nM palbociclib‐treated MCF‐7 cells and PalbR cells from 3 biological replicates (full length blots in Supporting Information S1, Figure 5G, where one membrane has been probed with all antibodies). Quantitation of (H) CDK1, (I) pCDK1^T161,^ and (J) CDK1 activity (calculated as the ratio of pCDK1^T161^ to total CDK1 expression) by densitometry from 3 biological replicates. Band intensity normalized to respective GAPDH in (H‐I) or respective CDK1 expression in (J). Data in (H‐J) analyzed by one‐way ANOVA with Tukey's multiple comparisons test; error bars = standard error of the mean.

To determine how MCF‐7 cells respond to acute versus long‐term palbociclib treatment, we performed RNA‐seq on MCF‐7 cells treated with vehicle or 48 h palbociclib, and palbociclib‐resistant PalbR cells (Figure 5C). Multidimensional scaling was applied to visualize the relationships between samples based on their gene expression profiles, revealing that both acute and prolonged palbociclib exposure drove distinct transcriptional profiles (Figure 5D and Table S1).

To identify pathways underlying these changes, we tested genes upregulated or downregulated more than onefold for over‐representation of Hallmark gene sets (Table S2). As expected, cell cycle pathways including G2/M CHECKPOINT and E2F TARGETS were the signatures most downregulated by acute palbociclib treatment (Figure 5E). These pathways were re‐engaged in PalbR cells – relative to acute palbociclib treatment, they were significantly upregulated, and relative to vehicle, they were no longer differentially expressed, indicating that palbociclib‐resistant cells restore proliferative transcriptional programs. Gene‐level analysis of REACTOME_CELL_CYCLE_MITOTIC (Figure 5F) confirmed this pattern across the pathway. Canonical mitotic drivers (*CDK1* and *CCNB1)* and upstream regulators (*CDC25A, CDC25B*, *CDC25C*, *WEE1, PKMYT1)* were strongly suppressed after 48 h palbociclib and restored to near vehicle‐like levels in PalbR cells. We also observed E2F regulated genes including *CDK4*, *CDK6*, and D‐ and E‐type cyclins, were similarly restored in PalbR cells relative to acute palbociclib treatment (Figure 5F). The partial restoration of proliferative genes parallels the recovery of proliferative capacity in PalbR cells, albeit at a slower rate than MCF‐7‐Vehicle cells (Figure S5B).

To further examine the re‐engagement of proliferation and validate the RNA‐seq findings, we characterized the expression of CDK1 and cyclin B1 in the resistant state. There was no significant difference in *CDK1* and *CCNB1* mRNA levels of MCF‐7‐Vehicle and PalbR cells (Figure S5E,F). Protein analysis also showed no significant difference in total CDK1 or pCDK1^T161^ expression, CDK1 activity, or cyclin B1 expression between the PalbR and MCF‐7‐Vehicle cells (Figure 5G–J and Figure S5G,H). Thus, palbociclib‐resistant cells, despite proliferating more slowly, have similar CDK1 expression and activity as treatment‐naïve vehicle‐treated cells.

Given that palbociclib‐resistant cells retained basal CDK1 expression and activity, we asked whether they were sensitive to CDK1 inhibition. We treated MCF‐7‐Vehicle and PalbR cells with the pre‐clinical compound RO‐3306, a specific CDK1 inhibitor [67]. IC_50_ values did not differ significantly between PalbR and MCF‐7‐Vehicle cells (Figure S5I). Furthermore, RO‐3306 significantly reduced the ability of PalbR cells to form colonies (Figure S5J,K), suggesting CDK1 as a therapeutic vulnerability in a CDK4/6 inhibitor‐resistant setting.

### 3.6 CDK1 is Upregulated at Disease Progression In Vivo and in Patient Tumors

Having established that CDK1 expression and activity are retained in palbociclib‐resistant cells in vitro, we next asked whether CDK1 activity is similarly elevated with disease progression in vivo and in patient tumors.

To address this we used the MMTV‐PyMT;MMTV‐Cre;Cdk1‐FRET biosensor mouse to examine tumors treated with the combination of tamoxifen and ribociclib (Figure 6A). Ribociclib was used in vivo as it has less autofluorescence than palbociclib and does not interfere with detection of the biosensor (data not shown). Treatment with tamoxifen + ribociclib led to a heterogenous response in mice, where we observed a markedly delayed time to disease progression in one mouse, which we classified as a partial responder (Figure 6B). Tumors that were unresponsive to the combination of tamoxifen + ribociclib were termed non‐responders, and tumors from these mice did not differ significantly to vehicle‐treated mice (Figure 6B). Using FLIM analysis to monitor Cdk1 activity in real‐time, we observed an increase in Cdk1 activity in tamoxifen + ribociclib non‐responsive tumors (Figure 6C). This increase was not observed in the partially responsive tumor when compared to vehicle‐treated tumors (Figure 6C). When quantified, the fluorescence lifetime was significantly lower in non‐responsive, resistant tumors (average lifetime 2.19 ns) compared to both vehicle‐treated and the partially responsive tumor (average lifetime 2.4 and 2.44 ns respectively), indicating elevated Cdk1 activity (Figure 6D), despite no difference in the rate of tumor growth. Given the small number of biologically independent mice and that comparisons were performed at the level of individual cells rather than tumors, these observations should be considered exploratory. To address this limitation, we next examined whether the same pattern was evident in a human cohort of CDK4/6 inhibitor‐resistant metastatic disease.

**FIGURE 6:**
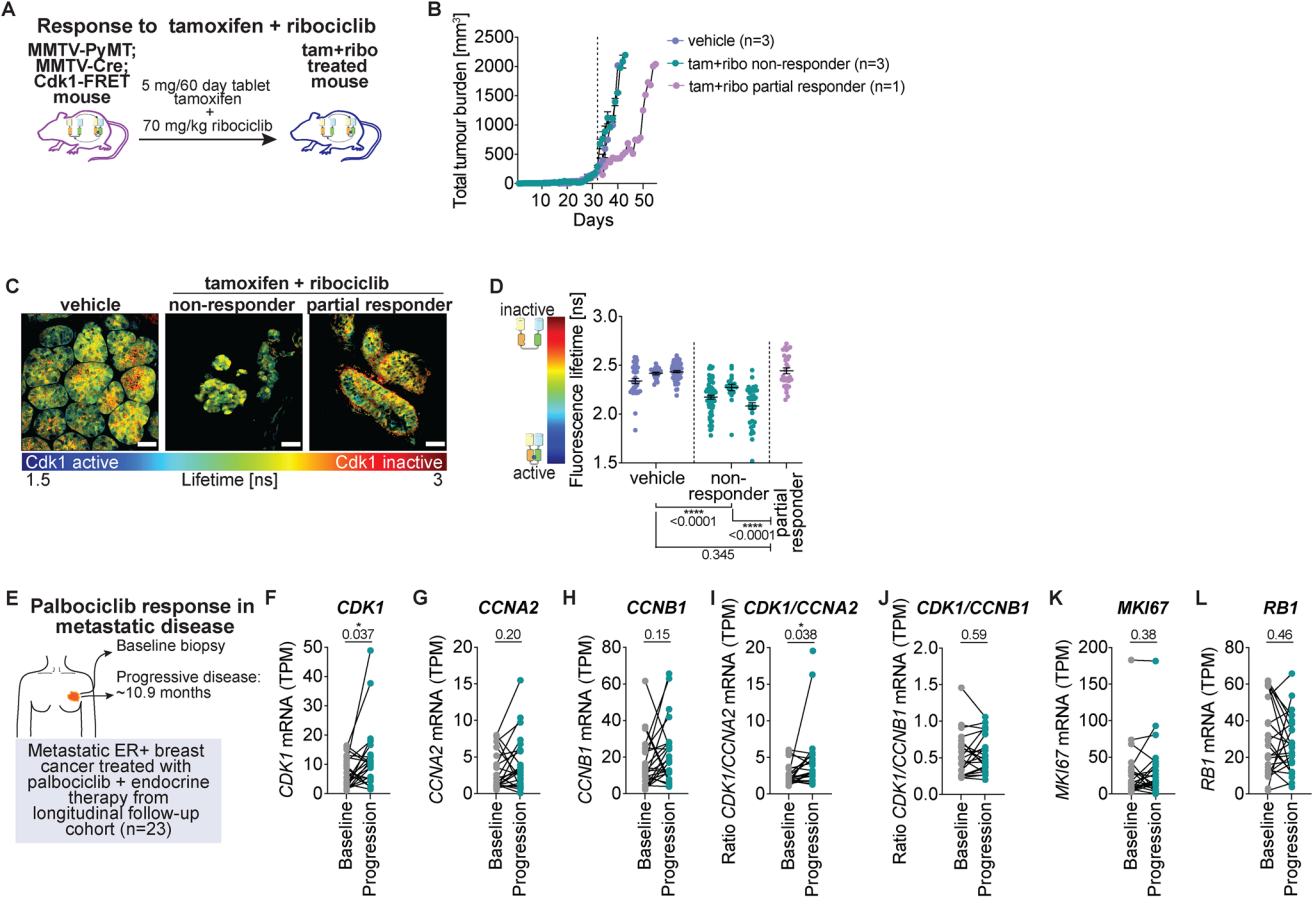
CDK4/6 inhibitor‐resistant non‐responsive tumors have elevated Cdk1. (A) Schematic of the in vivo generation of tamoxifen + ribociclib‐treated MMTV‐PyMT;MMTV‐Cre;Cdk1‐FRET (Förster Resonance Energy Transfer) tumors. (B) Total tumor burden of MMTV‐PyMT; MMTV‐Cre;Cdk1‐FRET mice treated with vehicle (0.5% methylcellulose; *n* = 3), treated with tamoxifen + ribociclib and non‐responsive (5 mg/60 day slow‐release tamoxifen tablet + 70 mg/kg ribociclib 5 days/week; *n* = 3), or treated with tamoxifen + ribociclib and partially responsive (5 mg/60 day slow‐release tamoxifen tablet + 70 mg/kg ribociclib 5 days/week; *n* = 1) for up to 23 days. Dotted line indicates treatment start. Data shown as mean ± standard error of the mean per group, or without error bars for the group with *n* = 1. (C) Representative images of Cdk1 activity in tamoxifen + ribociclib non‐responder (*n* = 3) and partial responder (*n* = 1) MMTV‐PyMT‐driven mammary tumors compared to a vehicle‐treated (*n* = 3) tumor. Scale bar = 50 µm. (D) Quantification of Cdk1 activity from data in (C). Data analyzed by one‐way ANOVA with Tukey's multiple comparisons test (n = 351 cells analyzed in total). (E) Schematic of a cohort of 23 metastatic estrogen receptor‐positive (ER+) breast cancer patients treated with palbociclib and endocrine therapy who go on to progressive disease [56]. mRNA expression of (F) *CDK1*, (G) *CCNA2*, (H) *CCNB1*, (I) *CDK1/CCNA2* ratio, (J) *CDK1/CCNB1* ratio, (K) *MKI67* and (L) *RB1* expression in biopsies taken at baseline and at disease progression. Difference between baseline and progressive disease analyzed by Wilcoxon matched‐paired signed‐rank test. TPM = transcripts per million.

We examined *CDK1* expression in tumors from a cohort of patients followed longitudinally during CDK4/6 inhibitor and endocrine therapy treatment (GSE186901) [56]. 23 patients in this cohort had been diagnosed with metastatic ER+ breast cancer (either *de novo* or recurrent) and went on to have progressive disease after an average of 10.9 months, despite treatment (Figure 6E). RNA‐seq of matched samples from the primary biopsy and progressive disease were analyzed (Figure 6F–L). We found that *CDK1* and the ratio of *CDK1/CCNA2* were significantly higher in tumors post progression in these patients (Figure 6F,I). By contrast *MKI67* did not change between the primary and recurrent biopsies (Figure 6K). We also noted that *RB1* expression was still retained in tumors that had progressed on treatment (Figure 6L). Therefore, the specific upregulation of *CDK1* at progression, without a concurrent increase in global proliferation markers like *MKI67*, suggests *CDK1* may be a superior biomarker of acquired resistance to endocrine therapy and CDK4/6 inhibition.

## 4 Discussion

The use of CDK4/6 inhibitors now spans the entire clinical landscape of ER+ breast cancer, ranging from early aggressive disease to post‐resistance settings [2, 3, 4, 5, 6]. Although CDK4/6 inhibitors are highly effective across these contexts, the ER remains the only established molecular marker for patient selection [68]. Given that CDK4/6 inhibitors are administered over periods of years, superior biomarkers are needed to refine patient selection and mitigate both clinical and financial toxicities. While markers such as RB, cyclin D1, p16, and Ki67 have been proposed, they have not consistently demonstrated a reliable association with clinical outcomes [3, 66, 69, 70]. There is a growing body of literature questioning the prognostic reliability of Ki67 in ER+ disease outside the neoadjuvant setting, where it is best used as a dynamic marker [61, 62, 71]. Consequently, we investigated CDK1, as its non‐redundant role in mitosis and proliferation could make it a more precise indicator for patient selection and therapeutic response. Although CDK1 is expressed throughout the cell cycle and peaks during G_2_/M, its activity is exquisitely controlled by specific phosphorylation events [60]. During the progression of S phase, sequential phosphorylation at Y15 and T161 primes CDK1 for activation at the appropriate mitotic checkpoint, and both events serve as high‐fidelity correlates of CDK1 activity. We address this in our study by using multiple measures of CDK1 activity, including the activation of endogenous CDK1/cyclin B1 complex [21] using a biosensor mouse and the status of the CDK1 activation site (T161) [60].

In this study, we identify CDK1 activity as a candidate marker of ER+ breast cancer progression and of the response to CDK4/6 inhibitors. By utilizing powerful biosensor imaging and extensive clinical cohorts, we demonstrate that CDK1 activity is upregulated during the transition to invasive disease (Figure 1 and Figure 2). Interestingly the very earliest lesions of hyperplasia in the biosensor mouse model had lower activity of Cdk1 (Figure 1), perhaps reflecting oncogene‐induced senescence that can occur in the earliest stages of mammary cancers [72]. In patients with matched samples of hyperplasia, DCIS and/or IDC, the pCDK1^T161^/total CDK1 ratio increases stepwise through progression, culminating in a significantly higher activity in IDC (Figure 1). In separate cohorts we then demonstrate for stage 1 cancers that higher CDK1 phosphorylation, and the surrogate measure of activity by *CDK1&cyclin expression*, are associated with poorer prognosis for patients with those tumors (Figure 2). These data suggest that CDK1 activity is a good candidate for investigation as a prognostic biomarker of high‐risk early ER+ breast cancer and the decision to administer CDK4/6 inhibitors.

CDK4/6 inhibitors are also used extensively in the advanced setting for breast cancer, both for *de novo* metastatic disease and for metastatic disease that recurs after endocrine therapy [73, 74]. The major challenges in these settings are the occurrence of intrinsic resistance, occurring in 10%–15% of cancers, and acquired resistance, which eventually develops in the majority of cancers. The NeoPalAna trial [55] exemplifies the situation of intrinsic resistance, and the samples collected therein can show differences between responders and non‐responders. While limited by the small sample size in the non‐responder cohort, our exploratory analysis shows that the CDK1 axis is dysregulated in non‐responders, with trends towards higher basal levels of *CDK1* in non‐responsive tumors, and a failure for *CDK1* to be downregulated following CDK4/6 inhibitor treatment (Figure 3). Our investigations at a molecular level demonstrate that sustained CDK1 activity is an indicator of the rebound response following CDK4/6 inhibition, and this is a good surrogate for continuing proliferation (Figure 4). This suggests that CDK1 status could serve as a biomarker for stratifying patients whose tumors are likely to fail standard‐of‐care endocrine and CDK4/6 inhibitor therapies. Furthermore, exploration of the adaptive response to CDK4/6 inhibitors in alternate model systems could lead to important insights for prolonged treatment response.

As well as *de novo* resistance, we identified a dynamic change in CDK1 activity in CDK4/6 inhibitor resistance models (Figure 5 and Figure 6). Notably, we found in both the non‐responsive tumors in the biosensor mouse model and patient samples that resistant disease had higher Cdk1 or *CDK1* activity even though the relative growth rate or other proliferative markers in the tumor had not changed. In the in vitro model, we found that CDK1 levels and activity were restored in resistance, despite the fact that resistant cells grew significantly slower than untreated cells. In our RNA‐seq analysis, transcripts encoding *CDK7* and its CAK partners cyclin H (*CCNH*) and MAT1 (*MNAT1*) were not differentially expressed between vehicle, acute palbociclib, and PalbR conditions (Table S2), although we cannot exclude post‐translational regulation of CAK activity. Given recent evidence that CDK7 inhibition has efficacy in CDK4/6 inhibitor‐resistant models and patient tumors [75, 76], direct interrogation of the kinases controlling CDK1^T161^ phosphorylation is an important priority for future work.

Overall, our work indicates that CDK1 activity has potential as a biomarker for CDK4/6 inhibitor usage. In these experiments, we have only considered models resistant to palbociclib or ribociclib, and patient cohorts treated with palbociclib. Additionally, as our cohorts are restricted to palbociclib‐treated patients, future studies incorporating abemaciclib and ribociclib‐treated cohorts could be performed to broaden the clinical relevance of our observations.

As a constitutively expressed gene and protein, CDK1 itself is unlikely to be sensitive enough to be prognostic. We found that the pCDK1^T161^ antibody was an effective tool in primary, *de novo* and advanced cohorts and model systems, and this gives the rationale to investigate its correlation with outcome in larger cohorts. We also considered the potential for CDK1 drug targeting following the development of CDK4/6 inhibitor resistance. In this study we used RO‐3306, a preclinical tool compound, to demonstrate that palbociclib‐resistant cells remain dependent on CDK1 activity; however RO‐3306 has no clinical utility as it is rapidly cleared from the bloodstream [77] which precluded in vivo evaluation. There are several pan‐CDK inhibitors with some activity against CDK1, such as flavopiridol and dinaciclib, but there are currently no specific CDK1 inhibitors that have been trialled in patients. First‐ and second‐generation pan‐CDK inhibitors have been extensively trialled in various cancer settings, but these have limited efficacy and considerable toxicity in patients, and are yet to transition successfully into a clinical setting [78, 79, 80].

While CDK4/6 inhibitors have transformed the treatment of ER+ breast cancer, the lack of precise predictive biomarkers remains a critical barrier to optimal patient care. Our findings position CDK1 activity as a candidate proliferation biomarker for aggressive disease progression and intrinsic resistance. Across the cohorts examined here CDK1 provides prognostic information that is complementary to Ki67, and outperforms Ki67 in specific ER+ endocrine‐treated settings. By validating that CDK1 phosphorylation peaks during the transition to invasive carcinoma and correlates with poor patient outcomes, we propose CDK1 as a promising candidate biomarker for the next generation of precision oncology. Implementing this biomarker could refine the clinical use of CDK4/6 inhibitors, addressing the urgent need to predict CDK4/6 inhibitor insensitivity and tailor therapeutic strategies for high‐risk populations.

## Author Contributions

This study was conceived by D.H., M.N., P.T., and C.E.C. S.A., M.N., and C.E.C. designed experiments. S.A., K.J.F., N.C.B., and M.N. collected and analyzed data, and S.A. and M.N. interpreted data. S.O. collated and annotated patient cohorts, K.J.F. and V.L. performed the immunohistochemistry, and E.K.A.M. and M.L.H. performed blinded scoring of patient cohorts. D.H., C.V., D.Str, and D.Ste developed the Cdk1‐FRET biosensor animal model. L.E. performed bioinformatic analysis of the RNA‐seq dataset. C.G.M., V.L., J.S., and T.D.T.L. collected in vivo data and were involved in animal husbandry. S.A. and C.E.C. drafted the manuscript with input from all authors. All authors read and approved the final manuscript.

## Funding

S.A. was supported by the Australian Government Research Training Program Scholarship; D.Str and D.Ste were supported by Cancer Research UK core funding to the CRUK Scotland Institute (A31287); D.H. was supported by a Cancer Institute NSW (CINSW) Early Career Research Fellowship (ECF011) and the National Health and Medical Research Council (NHMRC) Ideas Grant (2012837); P.T. was supported by an NHMRC Investigator Grant (2016930) and a National Breast Cancer Foundation (NBCF) grant (IIRS‐21‐2017); M.N. was supported by a CINSW Early Career Research Fellowship (ECF012), St Vincent's Clinic Foundation and an NHMRC Ideas Grant (1188208); C.E.C. was supported by a CINSW Career Development Fellowship (2020/CDF1071), the Lysia O'Keefe Fellowship and an NBCF grant (IIRS‐19‐054).

## Ethics Statement

All animal experiments and endpoints were conducted in accordance with the St Vincent's Precinct and GIMR AEC guidelines (ARA 19/06, 19/10, 19/13, 22/08, 22/09, 22/10, 25/10, 25/11, 25/12) and the UK Animals (Scientific Procedures) Act 1986 (Project License P4B6898D0) and EU Directive 2010, and in compliance with the Australian Code of Practice For Care and Use of Animals for Scientific Purposes. For clinical cohorts, ethics approval was granted by the Human Research Ethics Committee of St Vincent's Hospital (HREC SVH H94/080 and HREC 06336 SVH H00036) and the Ethics Committee of the Royal Prince Alfred Hospital (X05‐0115).

## Conflicts of Interest

P.T. receives reagents from Kadmon Inc., InxMed (consultant), Redx Pharma, Equilibre Biopharmaceuticals and Amplia Therapeutics. Under a licensing agreement between Amplia Therapeutics and GIMR, D.H., and P.T. (consultant) are entitled to milestone payments. C.E.C. has received honoraria from Lilly Australia. All other authors declare that they have no competing interests.

## Supporting information




**Supporting File 1**: advs77920‐sup‐0001‐Figures.pdf.

**Supporting File 2**: advs77920‐sup‐0002‐Table1.xlsx.

**Supporting File 3**: advs77920‐sup‐0003‐Table2.xlsx.

## Data Availability

The patient data presented in Figures 1, 2, 3 are not publicly available to protect patient privacy, and further inquiries regarding data available within the Article or Supplementary Figures can be made to the corresponding author: C.E. Caldon, email address: l.caldon@garvan.org.au. The Cdk1‐FRET biosensor mouse model will be available upon request to the corresponding author: P. Timpson, email address: p.timpson@garvan.org.au. RNA‐seq data supporting the findings of this study have been deposited into the NCBI GEO repository under accession code: GSE341330.
