# Supplementary material for: Tracking CDK1 Dynamics With a Novel Biosensor Mouse and Clinical Cohorts to Predict CDK4/6 Inhibitor Sensitivity in ER+ Breast Cancer
Source: Adv Sci (Weinh). 2026 Oct 11:e77920. Online ahead of print. doi: 10.1002/advs.77920 (PMC13655259; doi:10.1002/advs.77920)
Supplement: Supplementary file 1 [file ADVS-9999-e77920-s003.pdf]

SUPPLEMENTARY FIGURES FOR:

Tracking CDK1 dynamics with a novel biosensor mouse and clinical cohorts to predict CDK4/6 inhibitor sensitivity in ER+ breast cancer

Sarah Alexandrou[1,2], Kristine J Fernandez[2], Min Li Huang[1,3^], Ewan K A Millar[4,5^], Claire Vennin[1,2], Leila Eshraghi[1,2], Tan Dat Timothy Lam[2], Victoria Lee[2], Nyah C Brooks[2], Janett Stoehr[2], Cristian Guaman Mendez[2], Douglas Strathdee[6], David Stevenson[6], David Herrmann[1,2], Sandra O'Toole[2,7], Paul Timpson[1,2*#], Max Nobis[8*#], C Elizabeth Caldon[1,2*#]

^ equal contribution

* equal contribution

# corresponding author

1 St Vincent's Clinical School, Faculty of Medicine, UNSW Sydney, Sydney, NSW, Australia

2 Garvan Institute of Medical Research, Sydney, NSW, Australia

3 SydPath Department of Anatomical Pathology, St Vincent's Hospital, Sydney, NSW, Australia

4 Department of Anatomical Pathology, NSW Health Pathology, St George Hospital, Sydney, NSW, Australia

5 St George & Sutherland Campus, School of Clinical Medicine, UNSW Sydney, Sydney, NSW, Australia

6 Cancer Research UK Scotland Institute, Glasgow, Scotland, UK

7 School of Medical Science, Faculty of Medicine and Health, University of Sydney, Sydney, NSW, Australia

8 Manchester Cell-Matrix Centre, Division of Cell Matrix Biology and Regenerative Medicine, School of Biological Sciences, Faculty of Biology Medicine and Health, The University of Manchester, Manchester, United Kingdom

Supplementary Figure 1

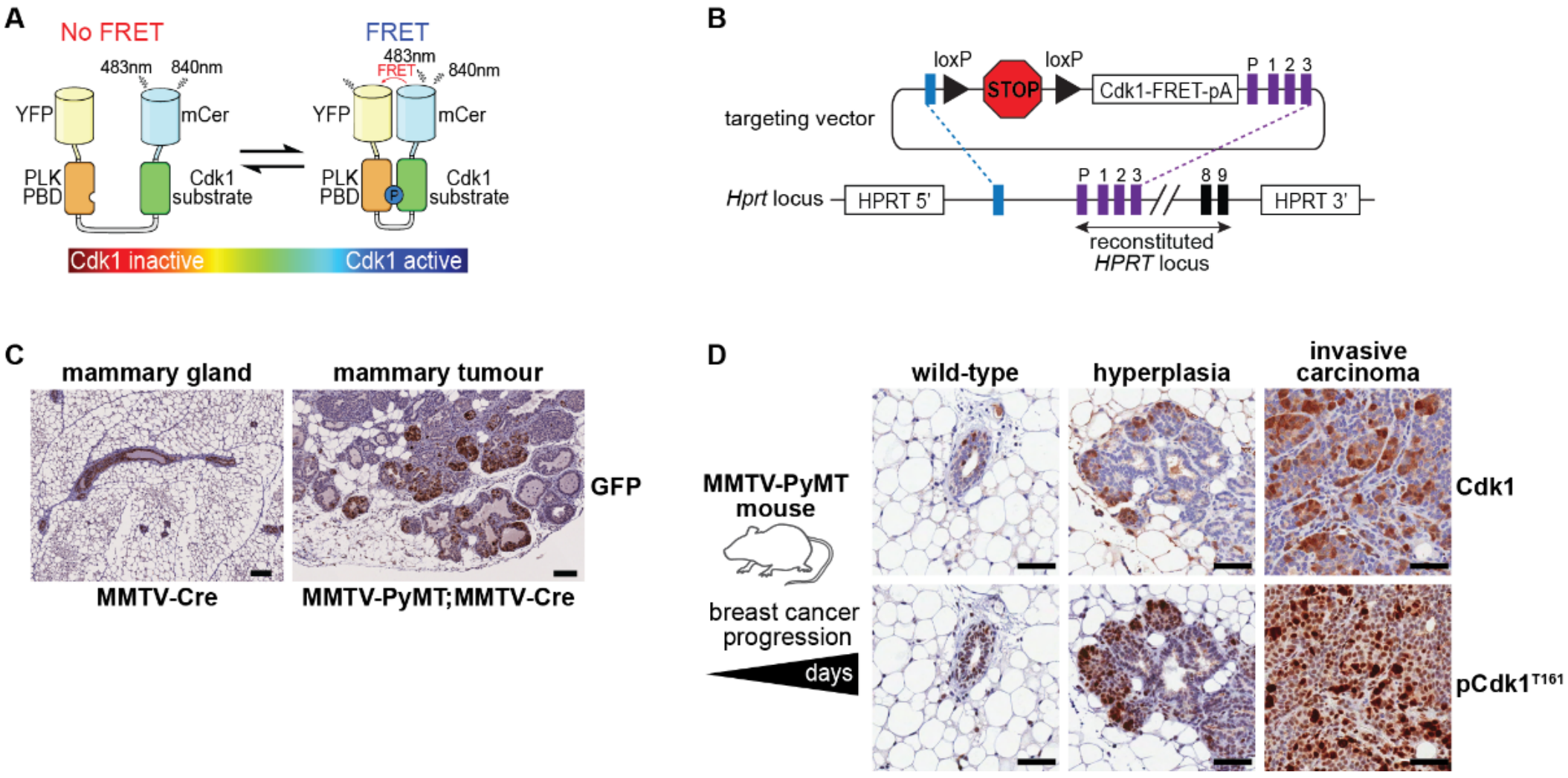

Supplementary Figure 1: Cdk1-FRET (Förster Resonance Energy Transfer) biosensor and Cdk1 activity in MMTV-PyMT carcinomas

(A) Schematic of the Cdk1-FRET biosensor[21], in Cdk1 inactive (non-FRET) and Cdk1 active (FRET) conformation. The biosensor consists of the donor fluorophore mCerulean (mCer) and the acceptor fluorophore yellow fluorescent protein (YFP). They are linked by a Polo-like kinase (PLK)-derived phospho-binding domain (PBD) and a 16aa autophosphorylation site of cyclin B1 (Cdk1 substrate). When Cdk1 is activated and bound to cyclin B1, it phosphorylates the biosensor at the Cdk1 substrate, resulting in a conformational change and an increased FRET. **(B)** *Hprt* targeting of the Cdk1-FRET biosensor, flanked by a lox-stop-lox domain allowing for tissue specific expression of the biosensor when crossed to a Cre driver line. **(C)** Representative GFP immunohistochemistry (IHC) of the Cdk1-biosensor in the mammary gland and mammary tumour of MMTV-Cre and MMTV-PyMT;MMTV-Cre mice. Scale bar = 100 µm. **(D)** Representative total Cdk1 and pCdk1^{T161} IHC of wild-type, hyperplasia and invasive carcinoma from MMTV-PyMT-driven mammary tumours. Scale bar = 50 µm.

Supplementary Figure 2

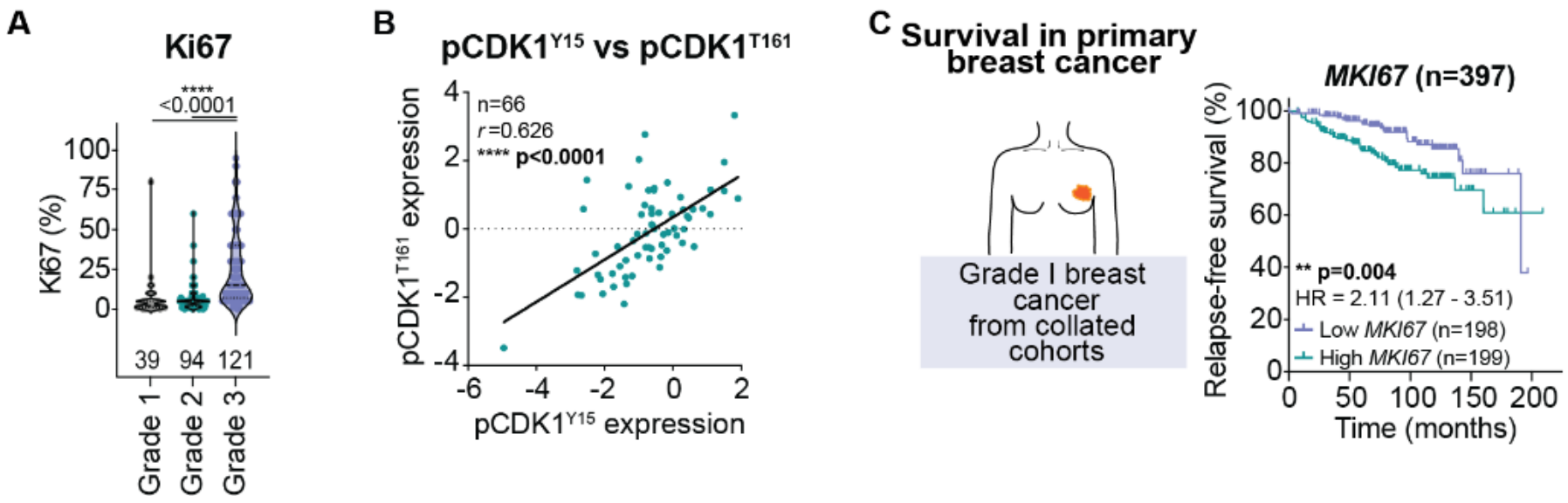

Supplementary Figure 2: Ki67 in early-stage breast cancer and CDK1 phosphorylation at Y15 and T161 phospho-sites

(A) Immunohistochemistry analysis of Ki67 in grade 1, 2 and 3 invasive ductal carcinomas from available cores from the Invasive Ductal cohort[36,38]. Each point represents one tumour; data analysed by Kruskal-Wallis test with Dunn's multiple comparisons test. **(B)** Correlation of $pCDK1^{Y15}$ and $pCDK1^{T161}$ in primary breast cancer from the CPTAC cohort (n=66). Pearson's correlation coefficient shown. **(C)** Relapse-free survival of patients with high and low *MKI67* in breast cancer patients with grade 1 disease from multiple combined cohorts accessed via KMPlotter[54]. Data was analysed by Log-rank (Mantel-Cox) test with hazard ratio.

Supplementary Figure 3

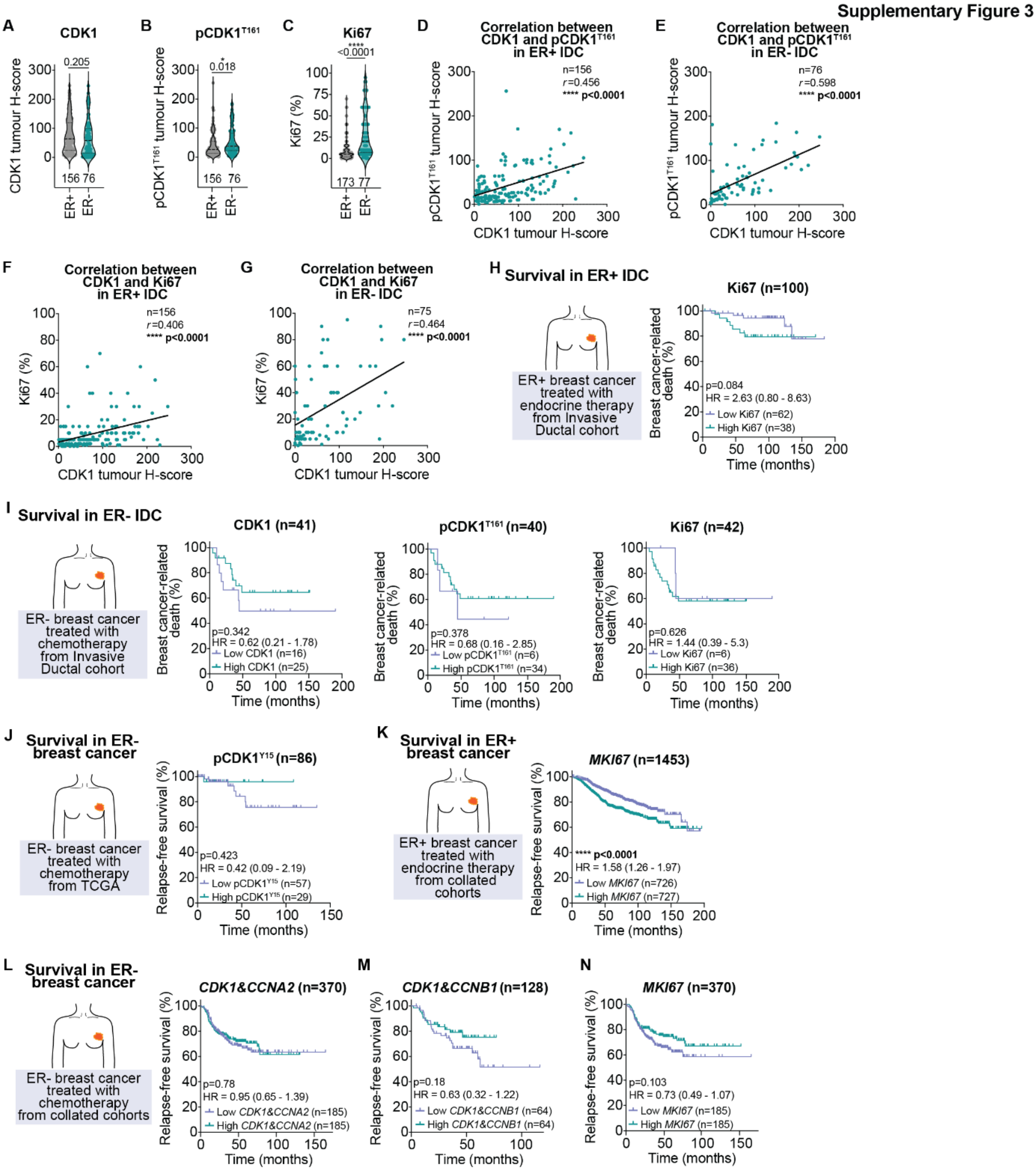

Supplementary Figure 3: CDK1 and Ki67 expression and survival across ER+ and ER- breast cancer

Immunohistochemistry (IHC) analysis of **(A)** total CDK1, **(B)** pCDK1^{T161} and **(C)** Ki67 in estrogen receptor positive (ER+) and negative (ER-) breast cancers from the Invasive Ductal cohort[36,38]. Data in **(A-C)** analysed by two-sided Mann-Whitney test. Correlation of total CDK1 and pCDK1^{T161} by IHC in **(D)** 156 ER+ and **(E)** 76 ER- invasive ductal carcinomas (IDC)[36]. Pearson's correlation

coefficient shown. Correlation of total CDK1 and Ki67 by IHC in **(F)** 156 ER+ and **(G)** 75 ER- IDC[36,38]. Pearson's correlation coefficient shown. **(H)** Breast cancer-related death in endocrine therapy-treated ER+ IDC split by high and low Ki67 from the Invasive Ductal cohort[36,38]. **(I)** Breast cancer-related death in chemotherapy-treated ER- IDC split by high and low total CDK1, pCDK1^{T161} and Ki67 from the Invasive Ductal cohort[36,38]. **(J)** Relapse-free survival of chemotherapy-treated ER- breast cancers split by high and low pCDK1^{Y15} from multiple combined cohorts accessed via KMPlotter[54]. **(K)** Relapse-free survival of endocrine therapy-treated ER+ breast cancers split by high and low *MKI67* from multiple combined cohorts accessed via KMPlotter[54]. Relapse-free survival of chemotherapy-treated ER- breast cancers split by high and low **(L)** *CDK1&CCNA2,* **(M)** *CDK1&CCNB1* and **(N)** *MKI67* from multiple combined cohorts accessed via KMPlotter[54]. Data in **(H-N)** analysed by Log-rank (Mantel-Cox) test with hazard ratio.

Supplementary Figure 4

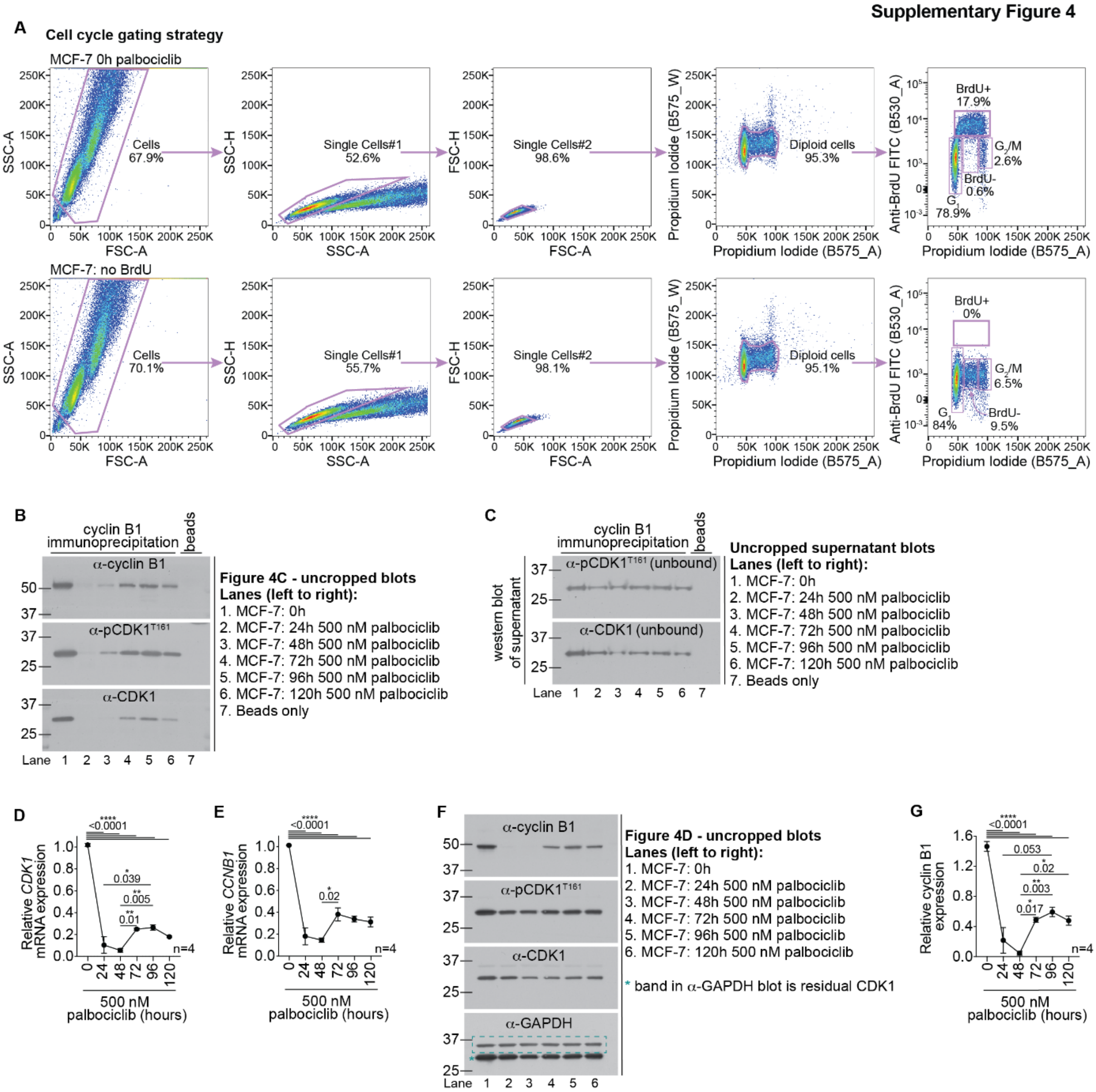

Supplementary Figure 4

H

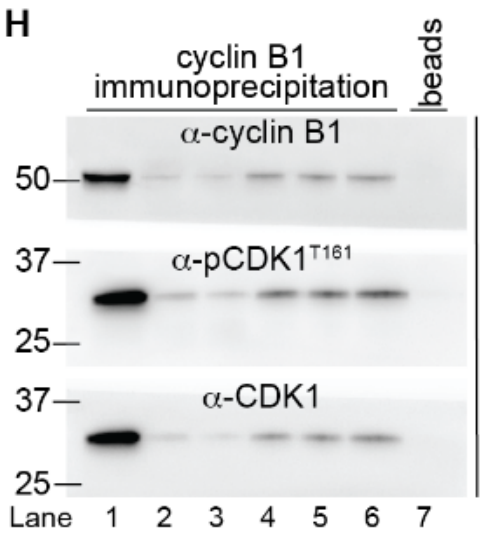

Figure 4H - uncropped blots
Lanes (left to right):
1. MCF-7: 0h
2. MCF-7: 24h 1 μM ribociclib
3. MCF-7: 48h 1 μM ribociclib
4. MCF-7: 72h 1 μM ribociclib
5. MCF-7: 96h 1 μM ribociclib
6. MCF-7: 120h 1 μM ribociclib
7. Beads only

I

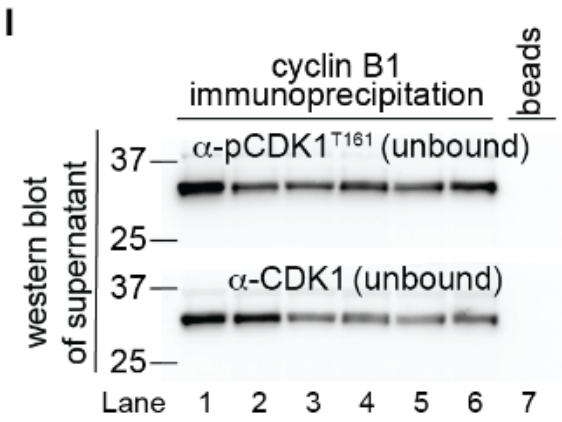

Uncropped supernatant blots
Lanes (left to right):
1. MCF-7: 0h
2. MCF-7: 24h 1 μM ribociclib
3. MCF-7: 48h 1 μM ribociclib
4. MCF-7: 72h 1 μM ribociclib
5. MCF-7: 96h 1 μM ribociclib
6. MCF-7: 120h 1 μM ribociclib
7. Beads only

J

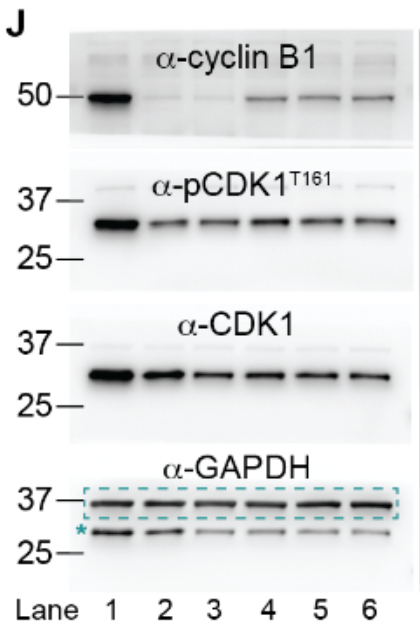

Figure 4I - uncropped blots
Lanes (left to right):
1. MCF-7: 0h
2. MCF-7: 24h 1 μM ribociclib
3. MCF-7: 48h 1 μM ribociclib
4. MCF-7: 72h 1 μM ribociclib
5. MCF-7: 96h 1 μM ribociclib
6. MCF-7: 120h 1 μM ribociclib

* band in α-GAPDH blot is residual CDK1

K

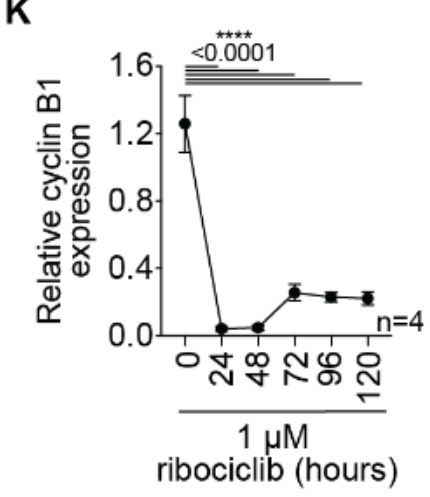

L

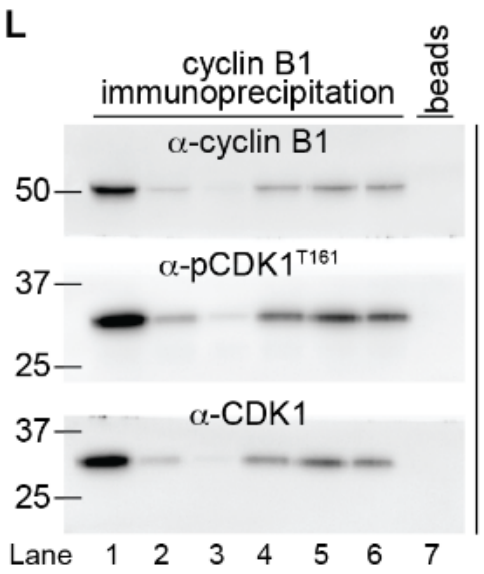

Figure 4M - uncropped blots
Lanes (left to right):
1. MCF-7: 0h
2. MCF-7: 24h 200 nM abemaciclib
3. MCF-7: 48h 200 nM abemaciclib
4. MCF-7: 72h 200 nM abemaciclib
5. MCF-7: 96h 200 nM abemaciclib
6. MCF-7: 120h 200 nM abemaciclib
7. Beads only

M

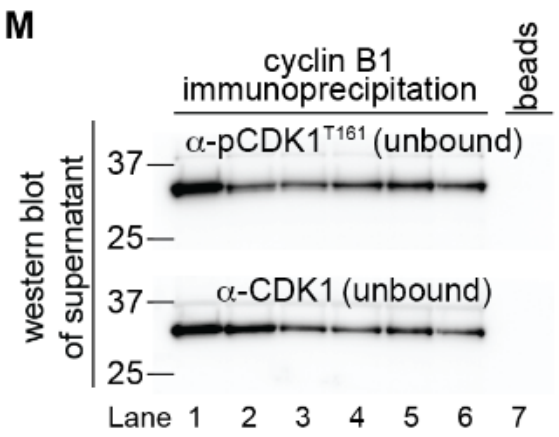

Uncropped supernatant blots
Lanes (left to right):
1. MCF-7: 0h
2. MCF-7: 24h 200 nM abemaciclib
3. MCF-7: 48h 200 nM abemaciclib
4. MCF-7: 72h 200 nM abemaciclib
5. MCF-7: 96h 200 nM abemaciclib
6. MCF-7: 120h 200 nM abemaciclib
7. Beads only

N

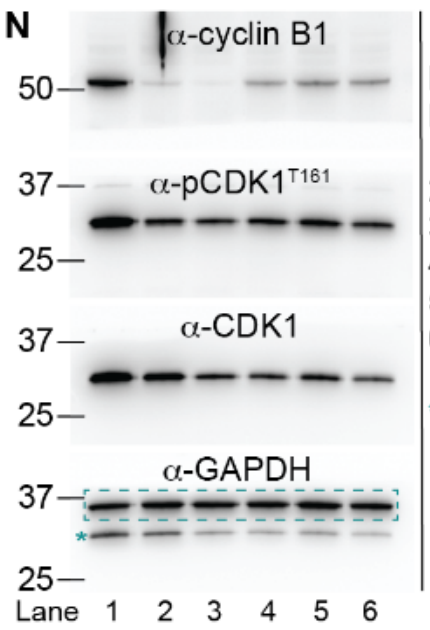

Figure 4N - uncropped blots
Lanes (left to right):
1. MCF-7: 0h
2. MCF-7: 24h 200 nM abemaciclib
3. MCF-7: 48h 200 nM abemaciclib
4. MCF-7: 72h 200 nM abemaciclib
5. MCF-7: 96h 200 nM abemaciclib
6. MCF-7: 120h 200 nM abemaciclib

* band in α-GAPDH blot is residual CDK1

O

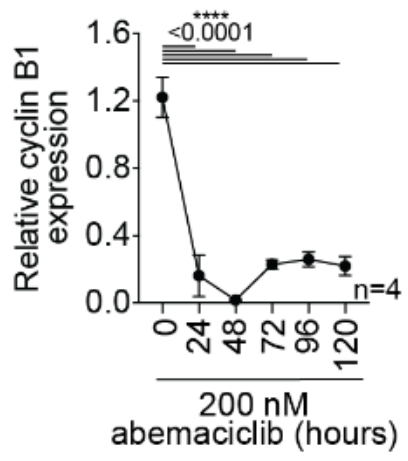

Supplementary Figure 4: Short-term response to CDK4/6 inhibitors is associated with recovery of CDK1 activity

(A) Gating strategy for bromodeoxyuridine (BrdU)/propidium iodide cell cycle analysis by flow cytometry as shown in Figure 4A. **(B)** Uncropped immunoprecipitation blots for Figure 4C. **(C)** Uncropped immunoprecipitation blots of unbound supernatant fraction. Quantitation of **(D)** *CDK1* and **(E)** *CCNB1* mRNA levels in MCF-7 cells treated with 500 nM palbociclib from 4 biological replicates. Expression normalised to *GAPDH*. **(F)** Uncropped Western blots for Figure 4D. **(G)** Quantitation of cyclin B1 by densitometry from 4 biological replicates. Band intensity normalised to respective GAPDH. **(H)** Uncropped immunoprecipitation blots for Figure 4H. **(I)** Uncropped immunoprecipitation blots of unbound supernatant fraction. **(J)** Uncropped Western blots for Figure 4I. **(K)** Quantitation of cyclin B1 by densitometry from 4 biological replicates. Band intensity normalised to respective GAPDH. **(L)** Uncropped immunoprecipitation blots for Figure 4M. **(M)** Uncropped immunoprecipitation blots of unbound supernatant fraction. **(N)** Uncropped Western blots for Figure 4N. **(O)** Quantitation of cyclin B1 by densitometry from 4 biological replicates. Band intensity normalised to respective GAPDH. Data in **(D-E, G, K, O)** analysed by one-way ANOVA with Tukey's multiple comparisons test, error bars = standard error of the mean. Polyvinylidene fluoride membranes in **(B-C, F, H-J, L-N)** were sliced into smaller sections to incubate with primary antibodies to allow accurate comparison of protein expression within a single sample. Full sections of the sliced membranes from each cropped Immunoprecipitation **(B-C, H-I, L-M)** or Western blot **(F, J, N)** are shown.

Supplementary Figure 5

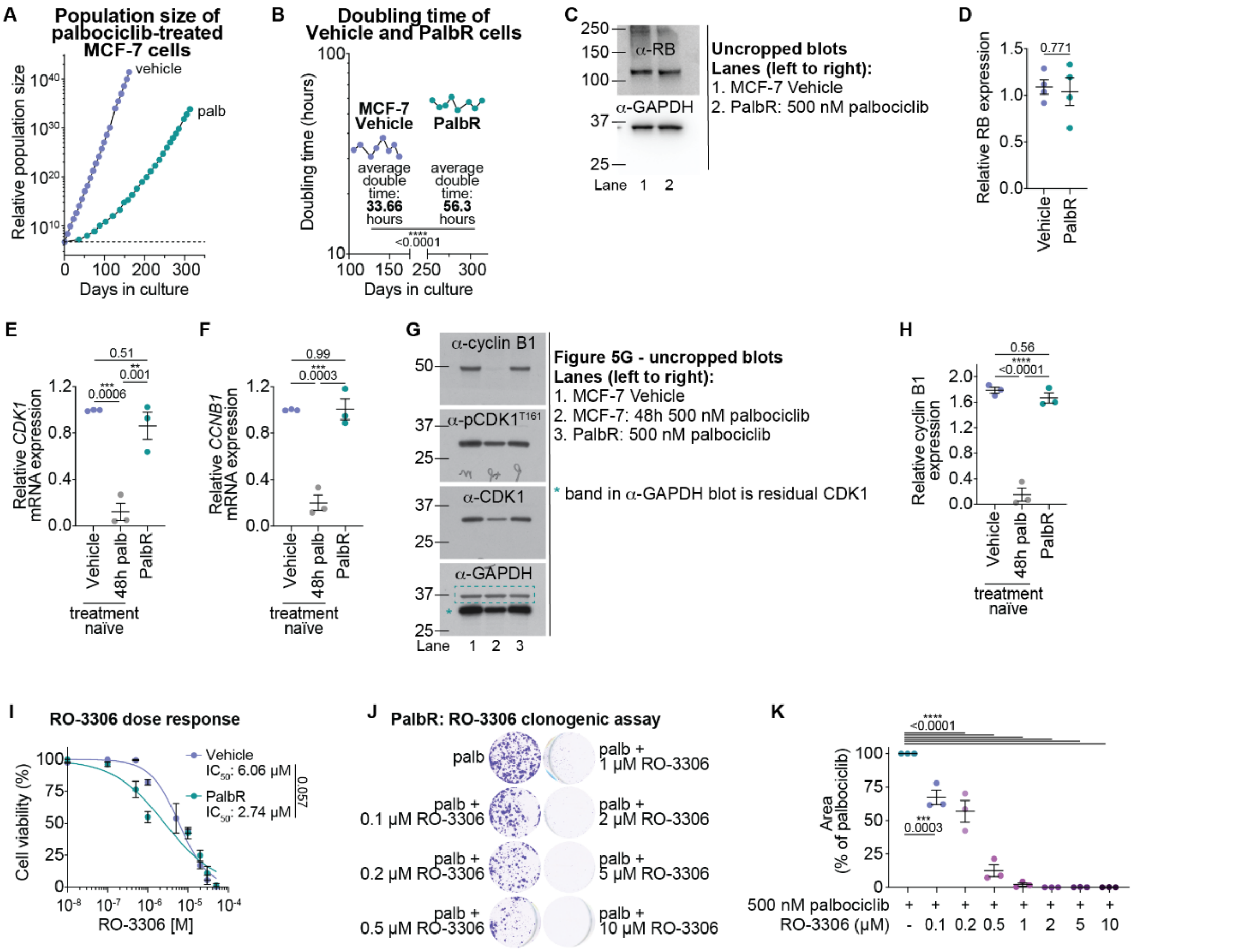

Supplementary Figure 5: Modelling CDK4/6 inhibitor resistance *in vitro*

(A) Population size of MCF-7 cells treated for up to 10.3 months with 500 nM palbociclib (palb) or vehicle (water). Dotted line indicates starting cell number. **(B)** Stable doubling time of PalbR and passage-matched vehicle-treated cell lines. **(C)** Representative uncropped Western blots of RB and GAPDH in MCF-7 Vehicle and PalbR cells from 4 biological replicates. **(D)** Quantitation of RB by densitometry from 4 biological replicates. Band intensity normalised to respective GAPDH. Quantification of **(E)** *CDK1* and **(F)** *CCNB1* mRNA levels in vehicle-treated and 48 hour 500 nM palbociclib-treated MCF-7 cells and PalbR cells from 3 biological replicates. Expression normalised to *GAPDH*. **(G)** Uncropped Western blots for Figure 5G. **(H)** Quantitation of cyclin B1 by densitometry from 3 biological replicates. Band intensity normalised to respective GAPDH. **(I)** Dose response curves of MCF-7 Vehicle and PalbR cells treated for 3 days with RO-3306 from 4 biological replicates. **(J)** Representative colony formation from 3 biological replicates of PalbR cells maintained in 500 nM palbociclib (palb) and treated with increasing concentrations of RO-

3306 for 2 weeks, with colony formation detected with 0.1% crystal violet stain. **(K)** Colony formation quantitated using ImageJ. Data in **(B, D, I)** analysed by two-tailed t-test and data in **(E-F, H, K)** analysed by one-way ANOVA with Tukey's multiple comparisons test, error bars = standard error of the mean. Polyvinylidene fluoride membranes in **(C, G)** were sliced into smaller sections to incubate with primary antibodies to allow accurate comparison of protein expression within a single sample. Full sections of the sliced membranes from each cropped Western blot **(C, G)** are shown.
